# Oncohistone interactome profiling uncovers contrasting oncogenic mechanisms and identifies potential therapeutic targets in high grade glioma

**DOI:** 10.1007/s00401-022-02489-2

**Published:** 2022-09-07

**Authors:** Robert Siddaway, Laura Canty, Sanja Pajovic, Scott Milos, Etienne Coyaud, Stefanie-Grace Sbergio, Arun Kumaran Vadivel Anguraj, Evan Lubanszky, Hwa Young Yun, Alessia Portante, Sheyenne Carette, Cunjie Zhang, Michael F. Moran, Brian Raught, Eric I. Campos, Cynthia Hawkins

**Affiliations:** 1 The Arthur and Sonia Labatt Brain Tumour Research Centre, Hospital for Sick Children, 686 Bay Street, Toronto, ON M5G 0A4 Canada; 2 Cell Biology Program, Hospital for Sick Children, Toronto, ON Canada; 3 Division of Pathology, Hospital for Sick Children, Toronto, ON Canada; 4 Princess Margaret Cancer Centre, University Health Network, 101 College Street, Toronto, ON M5G 1L7 Canada; 5 Université de Lille, Inserm, CHU Lille, U1192 - Protéomique Réponse Inflammatoire Spectrométrie de Masse - PRISM, 59000 Lille, France; 6 Department of Laboratory Medicine and Pathobiology, University of Toronto, Toronto, ON Canada; 7 Genetics & Genome Biology Program, Hospital for Sick Children, 686 Bay Street, Toronto, ON Canada; 8 Department of Molecular Genetics, University of Toronto, Toronto, ON Canada; 9 Department of Medical Biophysics, University of Toronto, Toronto, ON Canada; 10 Department of Paediatric Laboratory Medicine, Arthur and Sonia Labatt Brain Tumour Research Centre, University of Toronto, The Hospital for Sick Children, 555 University Avenue, Toronto, ON M5G 1X8 Canada

**Keywords:** H3K27M, H3G34R, pHGG, H3K9 methylation

## Abstract

Histone H3 mutations at amino acids 27 (H3K27M) and 34 (H3G34R) are recurrent drivers of pediatric-type high-grade glioma (pHGG). H3K27M mutations lead to global disruption of H3K27me3 through dominant negative PRC2 inhibition, while H3G34R mutations lead to local losses of H3K36me3 through inhibition of SETD2. However, their broader oncogenic mechanisms remain unclear. We characterized the H3.1K27M, H3.3K27M and H3.3G34R interactomes, finding that H3K27M is associated with epigenetic and transcription factor changes; in contrast H3G34R removes a break on cryptic transcription, limits DNA methyltransferase access, and alters mitochondrial metabolism. All 3 mutants had altered interactions with DNA repair proteins and H3K9 methyltransferases. H3K9me3 was reduced in H3K27M-containing nucleosomes, and cis-H3K9 methylation was required for H3K27M to exert its effect on global H3K27me3. H3K9 methyltransferase inhibition was lethal to H3.1K27M, H3.3K27M and H3.3G34R pHGG cells, underscoring the importance of H3K9 methylation for oncohistone-mutant gliomas and suggesting it as an attractive therapeutic target.

**Supplementary Information:**

The online version contains supplementary material available at 10.1007/s00401-022-02489-2.

## Introduction

Pediatric high-grade gliomas (pHGG) are the deadliest childhood cancers and are characterized by high frequency K27M mutations in histones H3.1 (H3.1K27M) and H3.3 (H3.3K27M), and G34R (or more rarely G34V/W) mutations in H3.3 (H3.3G34R) [49, 76, 84]. H3.3K27M is found in midline pHGG (~ 60% of cases), H3.1K27M is almost exclusive to the pons (~ 20%), while H3.3G34 mutations are almost exclusive to hemispheric pHGG (~ 10%) [61]. Age of onset also varies: H3.1K27M predominantly occurs in young children, H3.3K27M is most common in children but can occur at any age, and H3.3G34R occurs in adolescents and young adults [61]. These characteristics are suggestive of different cells or developmental stages of origin and different oncogenic mechanisms. Although first described in pHGG, histone mutations also occur in leukemia, sarcoma and chondroblastoma suggesting they have broader roles in oncogenesis beyond brain tumors [5, 6, 9, 11, 55, 60, 64].

Histone tail post-translational modifications play crucial roles in chromatin regulation. Polycomb-repressive complex 2 (PRC2)-mediated H3K27 trimethylation (H3K27me3) is associated with repressed chromatin, while H3K27 acetylation (H3K27ac) occurs in active promoters and enhancers [7]. H3.3K27M mutations have increased affinity for the EZH2 catalytic subunit of PRC2 and drive genome-wide H3K27me3 loss through dominant negative PRC2 inhibition and allosteric effects on PRC2 due to poor automethylation [10, 46, 54, 56]. Chromatin accessibility is further increased by H3K36 methylation and H3K27ac gains plus DNA hypomethylation, rendering it more permissive to transcription [10, 14, 16, 35, 52, 56, 65]. H3.3G34R mutations have more localized effects, inhibiting SETD2, to reduce cis-H3K36me2/3 and increase cis-H3K27me3, promoting tumorigenesis through alterations in chromatin looping that activate *PDGFRA* expression [13, 18, 44, 48, 56]. Although initial, direct effects on H3 marks at, or nearby, mutated amino acids have been identified, how oncohistones affect the broader chromatin environment and histone interaction landscape, and whether this can be exploited therapeutically, remains unknown.

To address this we generated unbiased, quantitative, proximity-dependent BioID interactomes of the H3.1K27M, H3.3K27M and H3.3G34R oncohistones plus their wild-type (WT) counterparts. This methodology circumvents issues arising from chromatin-extraction steps needed to affinity purify histones and their interactors [73]. All three mutations extensively mis-regulated the histone interactome, and identified key differences and commonalities in their oncogenic mechanisms. While H3K27M mutants had increased interaction with transcription factors (TFs), H3G34R lost TF interactions. Instead, its interactome suggested a mechanism related to increased cryptic transcription, loss of DNA methylation as well as metabolic changes. Notably, all three oncohistones had altered interactions with DNA repair proteins and H3K9 methyltransferases, which had altered enzymatic activity towards the oncohistones. Importantly, histone mutant primary patient pHGG cells were sensitive to inhibition of H3K9 methyltransferases, suggesting this could be an attractive therapeutic avenue for these deadly tumors.

## Materials and methods

### Ethical approval and patient samples

Patient material was collected after receiving informed consent, and work was approved by the Hospital for Sick Children Research Ethics Board (#1000055059).

### Reagents and plasmids

Doxycycline (Sigma; 10,000 × stock solution; 1 mg/ml) was resuspended in 70% ethanol. Biotin (BioShop and Sigma) was prepared as a 1000 × stock solution (50 mM) by dissolving 250 mg in 2 ml 30% v/v NH_4_OH on ice and neutralized by slowly adding 18 ml HCl (1 N), before sterile filtration through a 0.22 µm filter and storage at 4 °C. Chaetocin (Selleck) and OTS186935 (MedChemExpress) were dissolved in DMSO, aliquoted, and stored at − 80 °C.

H3.3 (*H3-3a*) or H3.1 (*H3C2*) cDNA that was WT or had K27M or G34R mutations was amplified from existing cDNA stocks and cloned between the XbaI/BamHI sites of a pCDH-CMV-MCS-EF1α-Hygro (SystemBioscience) previously modified to encode a FLAG/HA tag between the BamHI/NotI sites. K9M mutations were introduced with the Q5 site-directed mutagenesis kit (NEB). pcDNA5-FLAG-BirA* plasmids to create Flp-In T-REx HEK293 cells were generated as previously described [34]. Bacterial histone expression vectors were created by cloning cDNA into pET3a [17]. EHMT2 cDNA was amplified from pLenti6-MK1-EHMT2-V5 (Addgene #31113) and cloned between the KpnI and EcoRI sites of pFastBac (Invitrogen) to create pFastBac_EHMT2. SUV39H2 cDNA was amplified from a HEK293T cDNA library and inserted by Gibson assembly into pDest_pACE1 to create pACE1_SUV39H2. All plasmids were sequenced before use. shRNA targeting H3K9 methylases were from Sigma.

### Cell lines

Flp-In T-REx HEK293 (Thermo); HEK293T, U87, U343 (ATCC); MO3.13 (Cedarlane); iNHA [16]; and NHA (ABM) cells were cultured in DMEM (Gibco), supplemented with 10% FBS (Wisent) and 1% penstrep (Invitrogen), at 37 °C in 5% CO2. SF8628 DIPG cells were from Millipore and maintained in DMEM-High Glucose (Sigma), 10% FBS (EMD Millipore), 2 mM L-Glutamine (EMD Millipore) and 1X Penicillin–Streptomycin Solution (EMD Millipore). SU-DIPG-XIII, SU-DIPG-XVII, SU-DIPG-XXV, SU-DIPG-36 and SU-DIPG-50 cells were a kind gift from Michele Monje (Stanford University) [31]. H3.3G34R-mutant 7316-158 cells were from The Children’s Brain Tumor Tissue Consortium [43]. Primary HGG lines were cultured in Neurobasal(−A) media (Gibco) supplemented with B27(-A; Fisher-Gibco), growth factors (human-bFGF [20 ng/ml], human-EGF [20 ng/ml], human PDGF-AA [20 ng/ml], human PDGF-BB [20 ng/ml]; Shenandoah biotechnology inc) and heparin (10 ng/ml; Fisher/Stem Cell Technologies) on plates coated with Geltrex^™^ LDEV-Free Reduced Growth Factor Basement Membrane Matrix (Gibco) following the manufactures thin layer method (non-gelling). All cell lines were routinely tested for mycoplasma.

### Lentivirus

Plasmids were packaged into lentivirus by co-transfecting HEK293T cells with psPAX2 (Addgene#12260) and pMD2.G (Addgene#12259) using Lipofectamine 2000 (Invitrogen). Media was replaced the following day and viral supernatant collected 24 h later and concentrated using Lenti-X Concentrator (Clontech). Viral particles were resuspended in Optimem (Gibco) and stored at − 80 °C. Cells were transduced for 24 h. For MO3.13 and NHA cells, 10 μl/ml polybrene was added at the time of transduction (Santa Cruz).

### Cell viability assays

Cell viability assays were carried out by seeding cells in 6-well plates. For shRNA experiments, cells were transduced the next day, washed after 24 h, and harvested after 4 days. For drug experiments, the inhibitors or DMSO control was added the day after seeding and cells harvested after 4 days. Cells were analyzed by trypan blue exclusion assay with a Vi-CellXR (Beckman Coulter) or by CellTiter-Glo^R^ 2.0 assay (Promega).

Caspase activity assays were conducted by seeding cells in 96-well plates. After 24 h, cells were treated with chaetocin (125 nM) or DMSO and CellEvent Green Caspase-3/7 reagent (2 μM; Invitrogen). Cells were imaged and analyzed after 24 h using an Incucyte S3 Live-Cell Analysis System (Sartorius) using a 10 × objective.

### Immunofluorescence

Cells grown on coverslips to 80% confluence were washed, fixed in 4% PFA for 10 min, room temperature, and permeabilized (0.2% Triton X-100) for 10 min, room temperature. For mitotracker assays, 100 nM MitoTracker Red CMVRos (Invitrogen) was added for 20 min before fixation. Cells were blocked in 5% donkey serum (Jackson ImmunoResearch) and incubated overnight with primary antibodies Anti-HA tag antibody—ChIP Grade (abcam) and Anti-Histone H3.3 G34R Rabbit Monoclonal Antibody, Clone RM240 (RevMAb BioSciences). After 3 PBS-T washes Secondary antibodies (Fluorescein (FITC) AffiniPure Donkey Anti-Rabbit IgG; Jackson Immuno-Research Laboratories Inc.) were added for 1 h at room temperature. Coverslips were washed 3 times in PBS-T, and mounted with VECTASHIELD® Hardset Mounting Medium with DAPI (Vector Laboratories Inc.). Proximity ligation assays were carried out on coverslips permeabilized as above using Duolink In Situ Red Mouse/Rabbit kit (Sigma). Samples were imaged on a Leica Spinning Disk confocal microscope using a 40X lens. Analysis was carried out with ImageJ.

### Western blotting

Whole lysates were prepared in 2 × SDS-PAGE buffer (78.0 mM Tris [pH 6.8], 4% SDS, 20% glycerol, 100 mM DTT, 0.2% bromophenol blue) and separated on Novex Tris-Glycine 4–20% precast gels (Invitrogen). After transfer to PVDF membranes (Immobilon) that were then blocked in TBS-T containing 5% nonfat milk or BSA, primary antibodies were added overnight at 4 °C. After 3 TBS-T washes, membranes were incubated with HRP-conjugated secondary antibodies (BioRad, 1:10,000) and developed with ECL reagents (Pierce). Quantification was done with ImageJ.

### Immunoprecipitation

Co-immunoprecipitation assays were carried out by lysing cells in RIPA buffer (150 mM NaCl, 50 mM Tris [pH 8.0], 1% Triton X-100, 0.5% sodium deoxycholate, 0.1% SDS, supplemented with 1 × protease inhibitor cocktail [PIC]) on ice for 10 min and centrifugation at 14,000*g*, 10 min. After retaining an input fraction, the supernatant with incubated with 5 µg anti-HA overnight at 4 °C before Protein-G Dynabeads (Invitrogen) were added the next day for 2 h. Beads were washed three times in cold RIPA and boiled in hot 2 × SDS-PAGE buffer. Samples were analyzed by Western blotting

For nucleosome preparations, 40 million cells were suspended in buffer A (15 mM HEPES [pH 8.0], 4 mM MgCl_2_, 10 mM KCl, 1 mM β-mercaptoethanol, 1 mM PMSF, 1 × PIC) on ice for 5 min and NP-40 added to 0.2% for 5 min. Lysate was centrifuged at 600*g*, 5 min and the pellet washed in buffer A. The nuclear pellet was suspended in digestion buffer (15 mM HEPES [pH 8.0], 30 mM KCl, 1 mM CaCl_2_, 1 mM β-mercaptoethanol, 1 mM PMSF, 1 × PIC) and 40 units MNase (Worthington Biochemicals) added for 20 min at 37 °C. Digestion was quenched by addition of 2 × quenching buffer (10 mM EDTA [pH 8.0], 300 mM KCl, 0.2% Triton X-100) and samples centrifuged for 10 min at 14,000*g*. The supernatant was retained and aliquots taken for an input fraction and to check digestion. The remaining supernatant was incubated overnight with 5 µg anti-HA at 4 °C and Protein-G Dynabeads (Invitrogen) were added the next day for 2 h. Beads were washed three times in wash buffer (15 mM HEPES [pH 8.0], 150 mM KCl, 1 mM EDTA, 0.25% Triton X-100, 3 mM PMSF) and beads boiled in 2 × SDS lysis buffer.

### BioID

BioID experiments were performed by seeding Flp-In T-REx HEK293 cells to be ~ 60% confluent the next day. Cells were induced with 1 µg/ml sterile-filtered tetracycline in the presence of 50 µM sterile-filtered biotin for 24 h, washed and scraped in ice cold PBS and cell pellets snap frozen.

For streptavidin affinity purifications, five 15 cm plates of cells were prepared per sample. Cells were collected and pelleted (2000 rpm, 3 min), the pellet was washed twice with PBS and dried pellets were snap frozen. Cell pellets were resuspended in 10 ml of lysis buffer (50 mM Tris–HCl pH 7.5, 150 mM NaCl, 1 mM EDTA, 1 mM EGTA, 1% Triton X-100, 0.1% SDS, 1:500 protease inhibitor cocktail (Sigma), 1:1000 benzonase nuclease (Novagen) and incubated on an end-over-end rotator at 4 °C for 1 h, briefly sonicated to disrupt any visible aggregates, then centrifuged at 45,000*g* for 30 min at 4 °C. After retaining an input fraction, the remaining cleared supernatants were transferred to a fresh 15 mL conical tube. 30 μl of packed, pre-equilibrated streptavidin sepharose beads (GE) were added and the mixture incubated for 3 h at 4 °C with end-over-end rotation. Beads were pelleted by centrifugation at 2000 rpm, 2 min, and transferred with 1 ml of lysis buffer to a fresh Eppendorf tube. Beads were washed once with 1 ml lysis buffer and twice with 1 ml of 50 mM ammonium bicarbonate (pH 8.3). Beads were transferred in ammonium bicarbonate to a fresh centrifuge tube and washed two more times with 1 ml ammonium bicarbonate buffer. Tryptic digestion was performed by incubating the beads with 1 µg MS-grade TPCK trypsin (Promega, Madison, WI) dissolved in 200 μl of 50 mM ammonium bicarbonate (pH 8.3) overnight at 37 °C. The following morning, 0.5 μg MS-grade TPCK trypsin was added, and beads were incubated 2 additional hours at 37 °C. Beads were pelleted by centrifugation at 2,000*g*, 2 min, and the supernatant was transferred to a fresh Eppendorf tube. Beads were washed twice with 150 µL of 50 mM ammonium bicarbonate, and these washes were pooled with the first eluate. The sample was lyophilized and resuspended in buffer A (0.1% formic acid). 1/5th of the sample was analyzed per MS run.

### Mass spectrometry

Mass spectrometry was performed and analysed as previously described [34]. High performance liquid chromatography was conducted using a 2 cm pre-column (Acclaim PepMap 50 mm × 100 um inner diameter (ID)), and 50 cm analytical column (Acclaim PepMap, 500 mm × 75 um diameter; C18; 2 um; 100 Å, Thermo Fisher Scientific, Waltham, MA), running a 120 min reversed-phase buffer gradient at 225 nl/min on a Proxeon EASY-nLC 1000 pump in-line with a Thermo Q-Exactive HF quadrupole-Orbitrap mass spectrometer. A parent ion scan was performed using a resolving power of 60,000, then up to the twenty most intense peaks were selected for MS/MS (minimum ion count of 1,000 for activation) using higher energy collision induced dissociation (HCD) fragmentation. Dynamic exclusion was activated such that MS/MS of the same m/z (within a range of 10 ppm; exclusion list size = 500) detected twice within 5 s were excluded from analysis for 15 s.

### Metabolomic profiling

Cells snap frozen in liquid nitrogen were extracted in buffer (40% methanol, 40% acetonitrile, 2% formic acid) and incubated at 4 °C with shaking (1000 rpm) for 30 min before being centrifuged at 14,000 rpm at 4 °C. Supernatant was transferred to new tubes and metabolites pelleted with speed vacuum. The dry extracts were reconstituted in 70% acetonitrile containing internal standards (13C9 15N-tyrosine). Metabolite analysis was conducted with a TSQ Altis^™^ Triple Quadrupole Mass Spectrometer operating in the Multiple Reaction Monitoring mode (MRM), coupled with UltiMate 3000 HPLC system (Thermo Scientific) either by HILIC for anionic metabolites (sugar phosphates, organic acids, and nucleotides) or reverse phase column for amino acids.

For HILIC analysis, the metabolites were separated through a SeQuant^®^ ZIC^®^-pHILIC Guard (20 × 2.1 mm, 5 um; Millipore) and SeQuant^®^ ZIC^®^-pHILIC 5 µm polymer analytical column (150 × 2.1 mm, 5 um; Millipore) for both negative and positive MRM polarity modes. The mobile phase was composed of (A) 20 mM ammonium carbonate, pH 9.0 and (B) 100% acetonitrile, where the acetonitrile composition was 90–80% from 0 to 2 min; 80 to 70% from 2 to 4 min; 70 to 45% from 4 to 15 min; held for 4 min at 45% with flow rate at 0.15 ml/min; then subsequently returning within 11 min to 90% acetonitrile in mobile phase at flow rate 0.25 ml/min for column regeneration.

For reverse phase analysis, InertSustain PFP column (150 X 2.1 mm, 3um, GL Sciences, Inc. Japan) was used to perform positive mode MRM analysis. The mobile phase was composed of (A) 0.1% formic acid (B) acetonitrile, where the acetonitrile composition was 0% held for 4 min; 0 to 30% from 4 to 14 min; 30 to 40% from 14 to 16 min; 40 to 50% from 16 to 17 min; held for 3 min at 50%; then subsequently the gradient ramped back to 0% acetonitrile within 9 min at with flow rate at 0.2 ml/min to regenerate the column for the next run.

### Recombinant protein purification

#### Histone

pET3a-H3 plasmids were transformed into BL21(DE3) *E.coli* cells. Colonies were expanded in LB overnight and a 1 L culture inoculated, grown to OD600 = 0.4 and induced with 0.4 mM isopropyl β-d-1-thiogalactopyranoside (IPTG) for 4 h at 37 °C. Cultures were harvested by centrifugation at 3000×*g* and the pellets stored at − 80 °C.

Bacterial cell pellets expressing recombinant (untagged) histone proteins were Dounce homogenized 10–15 times in 10 × cell pellet volume of Histone Wash Buffer (50 mM Tris pH 7.5, 100 mM NaCl, 1 mM EDTA, supplemented with 1 mM DTT, 5 mM phenylmethylsulfonyl fluoride [PMSF]) with a tight pestle and subjected to three freeze–thaw cycles before treatment with 10 µL/mL of lysozyme (VWR) for 2 h on ice with occasional gentle mixing. The lysate was Dounce homogenized 10–15 times with a tight pestle and centrifuged for 20 min at 4 °C at 23,000×*g*. The pellet containing inclusion bodies was suspended in 100 mL Histone Wash Buffer supplemented with 1% Triton X-100, Dounce homogenized 10–15 times with tight pestle, and centrifuged at 25,000×*g* at 4 °C for 10 min. The process was repeated and the samples left to incubate in wash buffer at 4 °C overnight with gentle rotation. The inclusion bodies were re-centrifuged the next day, and the pellet washed twice more with 100 mL Histone Wash Buffer with 1% Triton X-100 and twice again with Histone Wash Buffer alone by repeating the dounce homogenization and centrifugation process. The final sample pellet was homogenized in 25 mL of Protein Unfolding Buffer (7 M guanidinium chloride, 20 mM Tris [pH 7.5], supplemented with 10 mM DTT) and gently stirred at 4 °C overnight. The samples were centrifuged at 25,000×*g* for 30 min at 20 °C, and the supernatant which contained the purified histone proteins was collected and stored at  − 80 °C.

The recombinant human H3.3 histone proteins and recombinant *Xenopus* H2A, H2B and H4 proteins were mixed in equimolar amounts. The mixture was dialyzed against Refolding Buffer (10 mM Tris [pH 7.5], 2 M NaCl, 20% glycerol, 1 mM EDTA, 5 mM β-mercaptoethanol) at 4 °C, with at least 3 changes of the buffer and one dialysis step performed overnight. Insoluble material was removed by centrifugation at 25,000 × g for 30 min at 4 °C. The supernatant contained the histone octamers and was aliquoted and stored at − 80 °C.

#### SUV39H2

pACE1_SUV39H2 plasmid was transformed into ArcticExpress (DE3) competent cells (Agilent). Colonies were expanded in LB overnight and a 1 L culture grown at 37 °C to OD600 = 0.4 before induction with 1 mM IPTG for 24 h at 10 °C. The cultures were harvested by centrifugation at 3000×*g* and the pellets stored at − 80 °C.

The harvested bacterial cell pellets were resuspended in 10 × pellet volume of lysis buffer (25 mM Tris [pH 7.6], 300 mM NaCl, 0.2 mM EDTA, 10% glycerol, 0.5% NP-40) and subjected to freeze–thaw and lysozyme treatment as described above. The lysate was sonicated (3 cycles of 30 s on, 30 s off, on ice, with mixing by gentle inversion in between) at an amplitude of 40 using a Misonix S-4000 Sonicator, and centrifuged at 25,000×*g* for 30 min at 4 °C to remove insoluble material.

Affinity purifications for StrepII-SUV39H2 were performed with StrepTrap HP (Cytiva). The lysate was bound onto the column, which was washed 2.5 × column volumes wash buffer 1 (20 mM Tris [pH 7.6], 100 mM NaCl, 0.2 mM EDTA, 20% glycerol), 2.5 × column volumes wash buffer 2 (20 mM Tris [pH 7.6], 300 mM NaCl, 0.2 mM EDTA, 20% glycerol, 0.05% NP-40) followed by 2.5 column volumes wash buffer 1. Protein was eluted in wash buffer 1 supplemented with 2.5 mM desthiobiotin. Elution fractions containing SUV39H2 were dialyzed against BC100 with 20% glycerol at 4 °C to remove desthiobiotin.

#### EHMT2

The generation of bacmids and baculoviral particles for EHMT2 was done according to the Bac-to-Bac Baculovirus Expression System (Invitrogen). 2 µg of bacmid DNA was transfected into *Spodoptera frugiperda* Sf9 cells using Effectene Transfection Reagent (QIAGEN) as instructed by the manufacturer’s protocol. The cells were incubated with the transfection mix for 6 h, with gentle agitation every 30–60 min at 27 °C. Media was replaced and cells incubated for 7–10 days at 27 °C, after which media was collected by centrifugation at 1000×*g* for 5 min at 4 °C and filtered with a 0.2 µm syringe filter. 10 ml baculovirus was used to infect 1 L Sf9 culture at 1–2 × 10^6^ cells/ml and grown at 27 °C with shaking at 110 rpm for 72 h. Cells were harvested by centrifugation at 1000×*g* for 10 min at 4 °C, washed in ice-cold PBS and stored at − 80 °C.

Pellets were resuspended in 5 pellet volumes of lysis buffer (20 mM Tris [pH 7.6], 5 mM KCl, 0.2 mM EDTA, 3 mM imidazole), subjected to one freeze–thaw cycle and sonicated for 3 cycles (30 s on, 30 s off with gentle mixing by inversion in between) at an amplitude of 40 with a Misonix S-4000 Sonicator. The lysate was then centrifuged at 25,000×*g* for 1 h at 4 °C to remove the insoluble material, and the supernatant retained.

Affinity purifications for EHMT2 was performed with the HisTrap HP column (Cytiva). The lysate was bound onto the column in lysis buffer, then washed with 2.5 × column volumes wash buffer 1 (20 mM Tris [pH 7.6], 100 mM NaCl, 0.2 mM EDTA, 20% glycerol, 30 mM imidazole), 2.5 × column volumes wash buffer 2 (20 mM Tris [pH 7.6], 300 mM NaCl, 0.2 mM EDTA, 20% glycerol, 0.05% NP-40) followed by 2.5 column volumes wash buffer 1. Protein was eluted with elution buffer (20 mM Tris [pH 7.6], 100 mM NaCl, 0.2 mM EDTA, 20% glycerol, 300 mM imidazole). Elution fractions containing EHMT2 were dialyzed against BC100 with 20% glycerol at 4 °C to remove imidazole.

### Histone methyltransferase assays

For HMT assays with EHMT2, 40 pmol of histone proteins were mixed with increasing amounts of EHMT2 enzyme (0, 0.09, 0.18, and 0.36 pmol) in buffer (50 mM Tris [pH 8.8], 10% glycerol, 5 mM MgCl_2_, supplemented with 0.5 mM DTT and 0.5 mM S-adenosyl methionine (SAM). The samples were incubated at 30 °C for 1 h with gentle shaking. The reaction was stopped by the addition of 2 × Laemmli buffer and boiling at 95 °C for 10 min, and analyzed by western blotting.

For HMT assays with SUV39H2, 40 pmol of histone proteins were mixed with increasing amounts of SUV39H2 enzyme (0, 15, 30, 60 pmol) in buffer (50 mM Tris [pH 8.5], 20 mM KCl, 10 mM MgCl_2_, 250 mM sucrose, 10 mM β-mercaptoethanol, and 0.5 mM SAM). The samples were incubated at 37 °C for 1 h with gentle shaking. The reaction was stopped and analyzed as described for EHMT2.

### Mass spectrometry analysis

#### BioID

For protein identification, Thermo.RAW files were converted to the mzXML format using Proteowizard [47], then searched using X!Tandem [21] and COMET [26] against the human Human RefSeq Version 45 database (containing 36,113 entries). Data were analyzed using the trans-proteomic pipeline (TPP) via the ProHits software suite (v3.3) [23, 41, 59]. Search parameters specified a parent ion mass tolerance of 10 ppm, and an MS/MS fragment ion tolerance of 0.4 Da, with up to 2 missed cleavages allowed for trypsin. Variable modifications of + 16@M and W, + 32@M and W, + 42@N-terminus, and + 1@N and Q were allowed. Proteins identified with an iProphet cut-off of 0.9 (corresponding to ≤ 1% FDR) and at least two unique peptides were analyzed with SAINT Express v.3.3.1. Ten control runs (from cells expressing the FlagBirA* epitope tag) were collapsed to the two highest spectral counts for each prey and compared to the two biological and two technical replicates of each histone BioID experiment. High confidence interactors were defined as those with Bayesian false discovery rate (BFDR) ≤ 0.01. Raw mass-spectrometry data is available for download from https://massive.ucsd.edu/, accession numbers MSV000087736 and MSV000089173.

#### Metabolomics

Standard compound mix was used to confirm the transitions and adjust the retention time.

Both internal standard and standard mix were used to monitor the instrument performance.

Pooled sample with different injection volumes were used for generating standard curve relative metabolites concentration analysis. Skyline (https://skyline.ms/project/home/software/Skyline/begin.view) [62] was used to integrate peak area and export peak intensity table or concentration quantification based on the standard curve.

MetaboAnalyst (http://www.metaboanalyst.ca/MetaboAnalyst/) [86] was used for metabolite statistical analysis and pathway analysis.

### Bioinformatics

Enrichment of CORUM protein complexes [29], Reactome and KEGG pathways, and Gene Ontology Cellular Compartments were tested using g:profiler [71]. Terms with adjusted *p* value < 0.05 were retained and networks constructed created with EnrichmentMap in Cytoscape v3.5.1 [77]. Node size and colour reflects term significance and edge weight the similarity between proteins or genes contributing to each node. Gene Ontology Biological Processes were tested with DAVID using Fisher’s exact test, retaining terms with adjusted *p* value < 0.05 [40].

### RNA-seq analysis

Previously published RNA-Seq datasets were retrieved to analyze H3K27M [69, 78] and H3.3G34R [18] mutant HGG. Quality-trimmed reads were aligned to human genome build GRCh37 using Trimmomatic and STAR [12, 25] and duplicates marked with Picard-tools. Gene level read counts were produced with HTSeq [3] and differentially expressed genes identified with DESeq and edgeR [2, 72]. Gene set enrichment analysis (GSEA) was used to determine enrichment of the NOTCH pathway [79].

Cryptic transcription was analyzed as described [63] using data from NSCs expressing H3.3WT or H3.3G34R Gene Expression Omnibus accession (GSE163044) [13]. Briefly, reads were trimmed using trim-galore (https://www.bioinformatics.babraham.ac.uk/projects/trim_galore) and aligned to human genome build GRCh37 using HISAT2 [50], and to the transcriptome using Salmon [70]. Exon expression levels in transcripts per million (TPM) were determined using featureCounts [57]. Subsequently, cryptic transcription was analyzed by calculating the ratios of internal exon expression to the first and second exon of the major transcript of multi-exon, expressed genes (TPM > 1) [63]. The log2-ratio of these ratios was then determined to compare cryptic transcription levels between H3.3G34R and WT.

### ChIP-seq analysis

Previously published [35] normalized read density for H3K27me3 and input samples from BT245 and G477 cell lines were downloaded from https://datahub-jv6f4mbl.udes.genap.ca and visualized in Integrated Genomics Viewer.

### DNA methylation analysis

Raw DNA methylation data was downloaded from Gene Expression Omnibus accessions GSE49822 and GSE55712 [10, 27]. Data were normalized with minfi [4]. No batch effects were observed in normalized data. Sample- and probe-level median beta values were calculated for each subgroup of samples. Differentially methylated regions (DMRs) were identified with the bumphunter function of minfi, requiring a minimum beta change of 0.25 and family-wise error rate < 0.05. DMRs were associated with genes using Homer [38].

### Statistical analysis

Unless otherwise stated, all tests are two-tailed Student’s *t* test, not assuming equal variance between samples, and were carried out in GraphPad Prism 8 or R-3.6.1.

### Data availability

This paper analyzes existing, publicly available data as outlined in the methods. Raw mass-spectrometry data is available for download from https://massive.ucsd.edu/, accession numbers MSV000087736 and MSV000089173.

## Results

### The oncohistone interactome

**Fig. 1:**
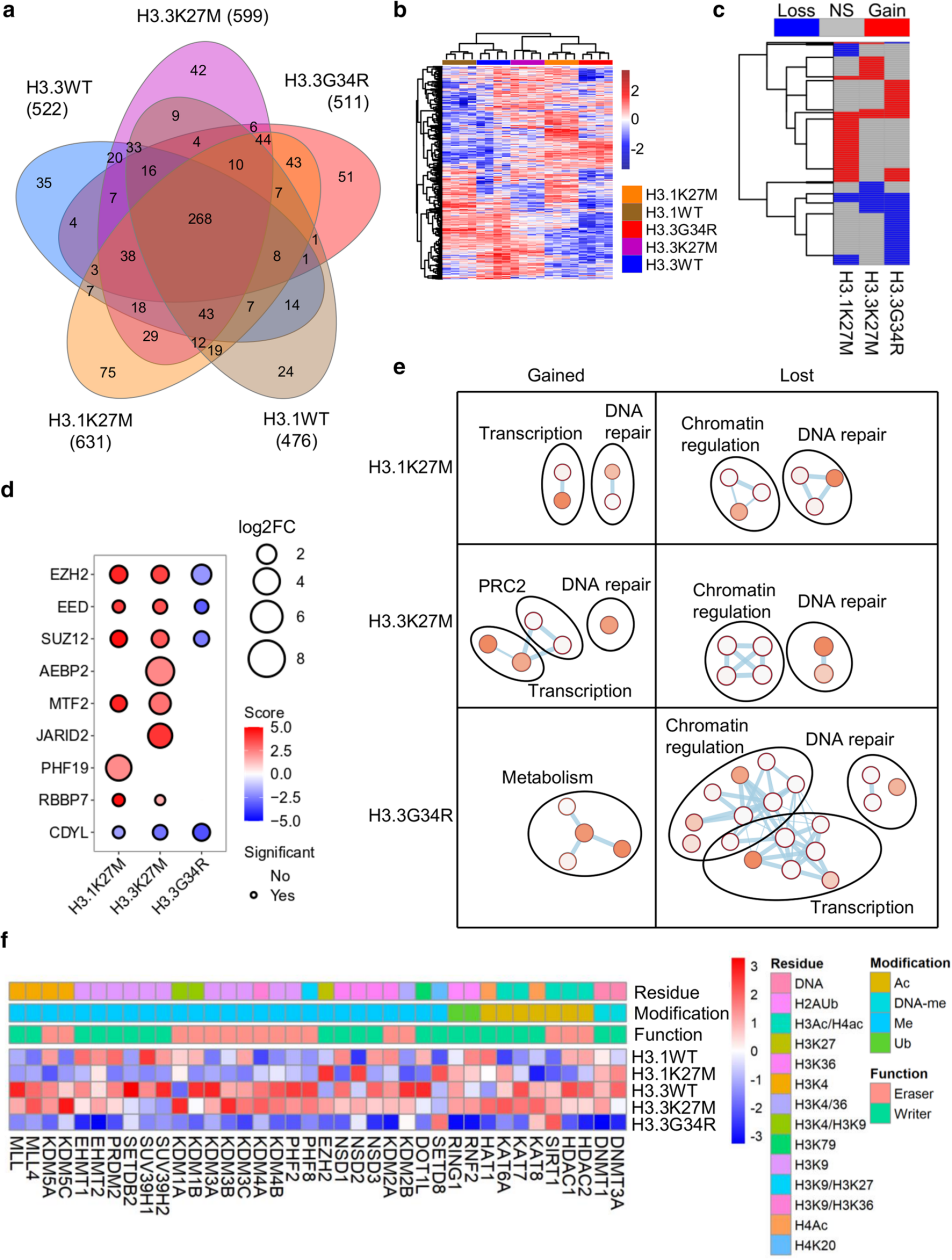
BioID interactome of wild-type and mutant H3. **A** Summary of high-confidence interactors detected with each histone. **B** Heatmap of all peptide counts of all high-confidence interactors identified in the experiment. **C** Heatmap depicting proteins differentially bound by any oncohistone relative to its WT control. **D** Bubble plot showing differential enrichment of PRC2 components with each oncohistone relative to its WT control. Size = log2(mutant/WT). Color represents significance. Statistically significant (*p* < 0.05) differential interactions have a black border. **E** Network of Reactome and KEGG pathways significantly enriched (*p* < 0.05) among differentially bound proteins by each oncohistone. Node size and color reflects significance and edge thickness reflects number of proteins shared between two nodes. **F** Heatmap of relative peptide counts of proteins with direct histone modifying activity identified by BioID. Average peptides per histone were Z-transformed

Doxycycline-inducible Flp-In T-Rex HEK293 cells expressing WT (H3.1WT, H3.3WT) or mutant (H3.1K27M, H3.3K27M, H3.3G34R) histone were established. 898 high-confidence interactors (mean 548, range 476-631) were identified by liquid chromatography-tandem mass-spectrometry (LC–MS/MS) following streptavidin pulldowns from cells induced in the presence of biotin. 268 common high-confidence interactors were shared by all H3 proteins (Fig. 1a; Table S1, online resource). Oncohistones had clear differences in global interactions, separating from WT histone in both hierarchical clustering and principal components analysis (PCA; Fig. 1b, Supp. Fig. S1a, b, online resource). As expected, based on their different functions there were some differences in interactions between the H3 variants H3.1WT and H3.3WT (~ 10% of the proteins). However, an oncohistone mutation resulted in a larger change in the histone interactome (range: 18–34%; Supp. Fig. S1c, online resource). 412 proteins were differentially bound by at least one oncohistone (Fig. 1c; Supp. Fig. S1d, online resource), of which 302 were unique to one mutant, 79 were differentially bound by two mutants (including the H3K36 dimethylase NSD1 and the H3K9 trimethylase SUV39H2) and 31 by all three (including EZH2, SUZ12, and the H3K9 trimethylase SUV39H1). Overall, gained interactions were most frequent for H3.1K27M while lost interactions were most frequent for H3.3G34R (Fig. 1c).

Initially, we confirmed the known increased binding of H3.3K27M to PRC2 in our dataset as well as by proximity ligation assays (PLA; Supp. Fig. S1e, online resource). Both K27M mutants had increased association with core PRC2 proteins (EZH2, EED, SUZ12) as well as RBBP7, an important PRC2 cofactor [36, 80] (Fig. 1d). PRC2 exists in at least two subcomplexes [36, 80]. PRC2.1 contains one of PHF1/PHF19/MTF2, which recruit PRC2.1 to chromatin; and EPOP/PALI1, which regulate transcription and stimulate PRC2 activity, respectively [89]. PRC2.2 contains AEBP2/JARID2, which both stimulate PRC2, while JARID2 also recruits PRC2.2 to chromatin. H3.3K27M had increased interaction with AEBP2 and JARID2 (PRC2.2) while H3.1K27M had increased interaction with PHF19 (PRC2.1) (Fig. 1d). MTF2, which is primarily responsible for PRC2.1 chromatin recruitment in embryonic stem cells [68], and is mutually exclusive with PHF19, was gained by both H3.1K27M and H3.3K27M. Overall, H3.3K27M gained interactions with both PRC2.2 and PRC2.1, and H3.1K27M gained interactions with PRC2.1. This may reflect the different modes of deposition of H3.1 and H3.3 into chromatin, with H3.3 expressed throughout the cell cycle and deposited into active chromatin as well as heterochromatin, while H3.1 is expressed and deposited in S-phase [30, 85]. Furthermore, CDYL, which bridges PRC2 to neighboring nucleosomes and is important for H3K27me3 spreading [91], lost interaction with the mutant histones, in keeping with reduced H3K27me3 spreading in H3K27M mutant cells (Fig. 1d).

H3.3G34R inhibits SETD2 and affects local H3K36me3 [44]. While SETD2 was not identified in our BioID experiment, the H3K36 dimethylases NSD1-3 had reduced binding to H3.3G34R, which we confirmed by PLA (Fig. 1f; Supp. Fig. S1f, online resource). H3K36me3 is antagonistic towards EZH2, and H3K27me3 is increased on H3.3G34R-mutant histones [13, 18, 44, 48, 56]. Paradoxically, BioID showed that H3.3G34R had reduced interaction with PRC2, suggesting that PRC2 may have reduced dwell time on H3K27 in H3.3G34R histones.

We next analyzed Reactome and KEGG pathways to understand how oncohistones remodel the histone interactome (Fig. 1e; Supp. Fig. S2a, online resource). The lost interactors of all 3 oncohistones were enriched for chromatin regulators. There were extensive variations in binding to 38 proteins with direct chromatin-modifying (either histone or DNA) enzymatic activity. Consistent with pathway analysis, H3.3G34R had the largest effects (Fig. 1f). Specifically, oncohistones had altered binding to H3K4, H3K9, H3K27 and H3K36 methylases and demethylases; histone acetyltransferases and deacetylases; and DNA methyltransferases; suggesting broader epigenetic effects than previously discovered.

Several noteworthy patterns emerged from this data. First, DNA methyltransferases and HDAC1/2 had reduced interactions with H3.3G34R, but not H3.1K27M/H3.3K27M (Fig. 1f). Second, as a family, H3K9 methylases had reduced interactions with all 3 oncohistones. H3.3G34R additionally lost interactions with H3K9 demethylases (Fig. 1f). Third, transcription was upregulated among H3K27M-specific proteins but downregulated among H3.3G34R-specific proteins (Fig. 1e, S2a). Fourth, DNA damage repair processes were both gained and lost with H3.1K27M and H3.3K27M, but primarily lost with H3.3G34R (Supp. Fig. S2b, online resource), suggesting that G34R-mutant tumors may have increased susceptibility to DNA damage, or impaired signaling to repair processes. Fifth, it was striking that all enriched pathways for H3.3G34R related to metabolism (Fig. 1e; Supp. Fig. S2a, online resource). Collectively, they suggest that H3K27M mutants reduce H3K27me3, permitting increased access to transcriptional machinery, while H3G34R has more local effects leading to altered metabolism and loss of DNA methylation.

### H3.3G34R localizes to mitochondria and is associated with altered mitochondrial metabolism

**Fig. 2:**
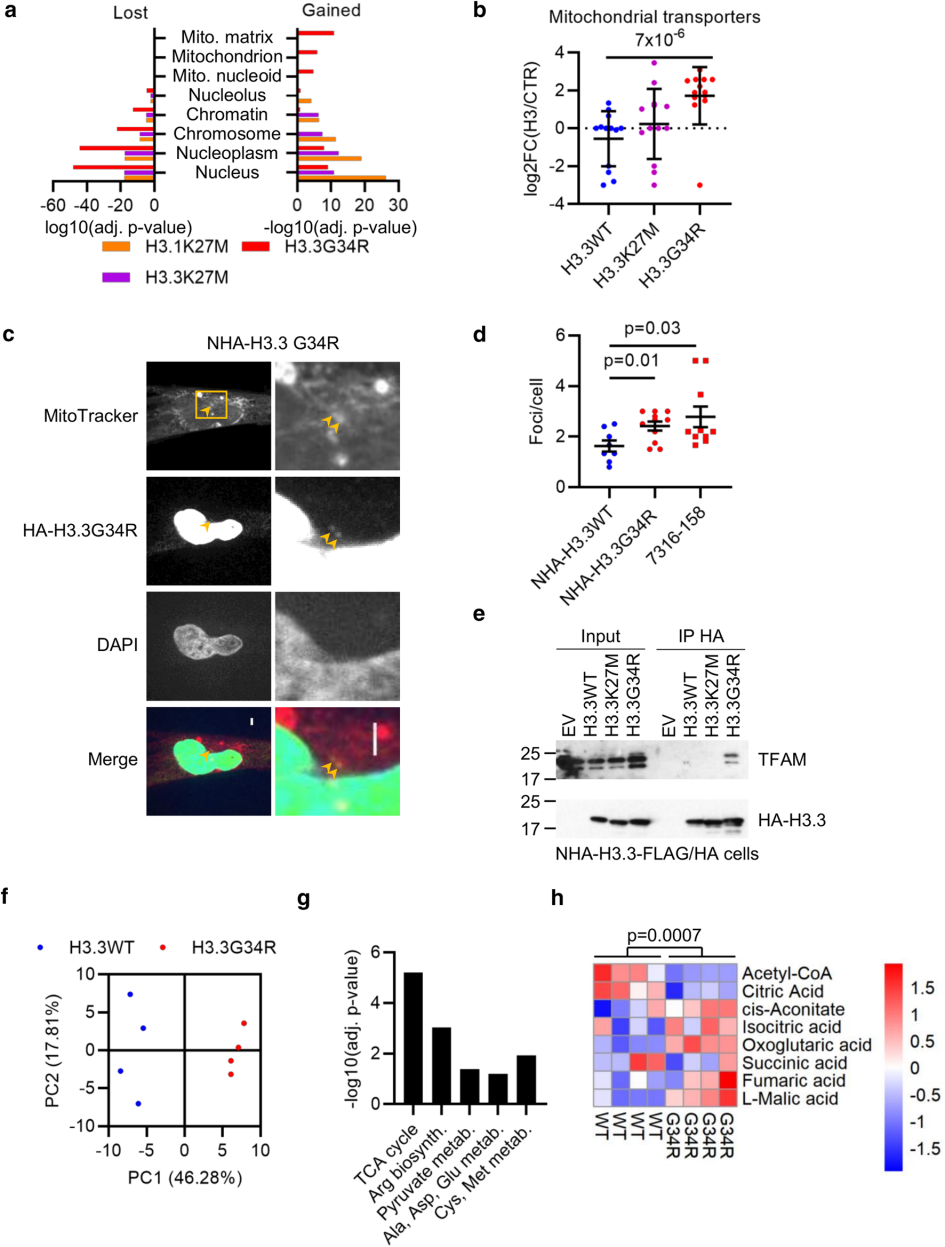
Mitochondrial localization of H3.3G34R. **A** Enrichment of subcellular localizations (Gene Ontology Cellular Compartments) among the proteins differentially bound by each oncohistone. **B** Relative enrichment [log2(H3/control)] of outer (TOMM) and inner (TIMM) mitochondrial transporter proteins associated with each histone. *p*: *t* test. **C** Confocal microscopy of NHA cells expressing H3.3G34R-FLAG/HA and stained with MitoTracker Red and HA. Box shows zoom area at right. Arrows mark co-localization of H3.3G34R with mitochondria. Scale bar: 3 μm. **D** Quantification of colocalization between MitoTracker Red and H3.3 in NHA cells expressing H3.3WT or H3.3G34R (antibody: HA tag) or 7316-158 cells (antibody: H3.3G34R). Bars show mean ± standard error of at least 7 fields of view. **E** Co-immunoprecipitation of H3-FLAG/HA and TFAM from NHA cells transduced with indicated constructs and subjected to HA IP. **F** Principal components analysis of normalized metabolite concentrations in NHAs expressing H3.3WT or H3.3G34R as determined by LC–MS (*n* = 4). **G** Enriched metabolic pathways in H3.3G34R vs H3.3WT NHAs. **H** Relative concentration of indicated TCA metabolites in H3-expressing NHA cell lines (*n* = 4). *p*: *t* test

Metabolic pathway enrichment, specifically among H3.3G34R-gained interactors (Fig. 1e; Supp. Fig. S2a, online resource), was unexpected. Nuclear proteins were enriched among all gained and lost interactors, while mitochondrial locations were exclusive to H3.3G34R-gained proteins (Fig. 2a; Supp. Fig. S3a, online resource), suggesting that H3.3G34R might localize to mitochondria. Transporter complexes spanning the outer and inner membranes mediate mitochondrial protein translocation [67]. Surprisingly, both outer (TOMM) and inner (TIMM) mitochondrial membrane transporters had increased association with H3.3G34R compared with H3.3K27M and H3.3WT (*p* ≤ 0.01; Fig. 2b; Supp. Fig. S3b, c, online resource).

To test whether H3.3G34R localizes to mitochondria, we co-stained normal human astrocytes (NHA) expressing H3.3G34R-FLAG/HA with MitoTracker Red, a mitochondrial stain. As expected, the vast majority of H3.3G34R was nuclear, but with focal accumulation in regions colocalizing with mitochondria (Fig. 2c). We also stained patient-derived H3.3G34R-mutant 7316-158 pHGG cells [43], finding colocalization of endogenous H3.3G34R with MitoTracker Red-labeled mitochondria (Supp. Fig. S3d, online resource). The number of colocalizing foci in NHA-H3.3G34R and 7316-158 cells was significantly greater than that in NHA-H3.3WT cells, suggesting that G34R mutant H3.3 is more likely to localize to mitochondria than WT H3.3 (Fig. 2d). Finally, mitochondrial transcription factor 1 (TFAM) was exclusively associated with H3.3G34R by both BioID and co-immunoprecipitation (Fig. 2e; Supp. Fig. S3e, online resource). TFAM regulates mitochondrial copy number, coats mitochondrial DNA to aid in its compaction, and activates mitochondrial promoters.

This localization with mitochondria and mitochondrial transcription factors led us to hypothesize that H3.3G34R may have metabolic effects on the cells. To test this, we employed LC–MS/MS to generate metabolomic profiles of NHAs expressing H3.3WT or H3.3G34R. H3.3 mutation status clearly separated cells in PCA of the 116 metabolites identified (Fig. 2f). The citric acid (TCA) cycle was the most enriched metabolic pathway among differentially accumulated metabolites in H3.3G34R mutant cells, indicating a higher level of mitochondrial metabolism in these cells (Fig. 2g, h). Overall, these data suggest that H3.3G34R more frequently localizes to mitochondria and may influence mitochondrial metabolism.

### H3.3G34R loses association with DNA methyltransferases leading to global DNA hypomethylation

**Fig. 3:**
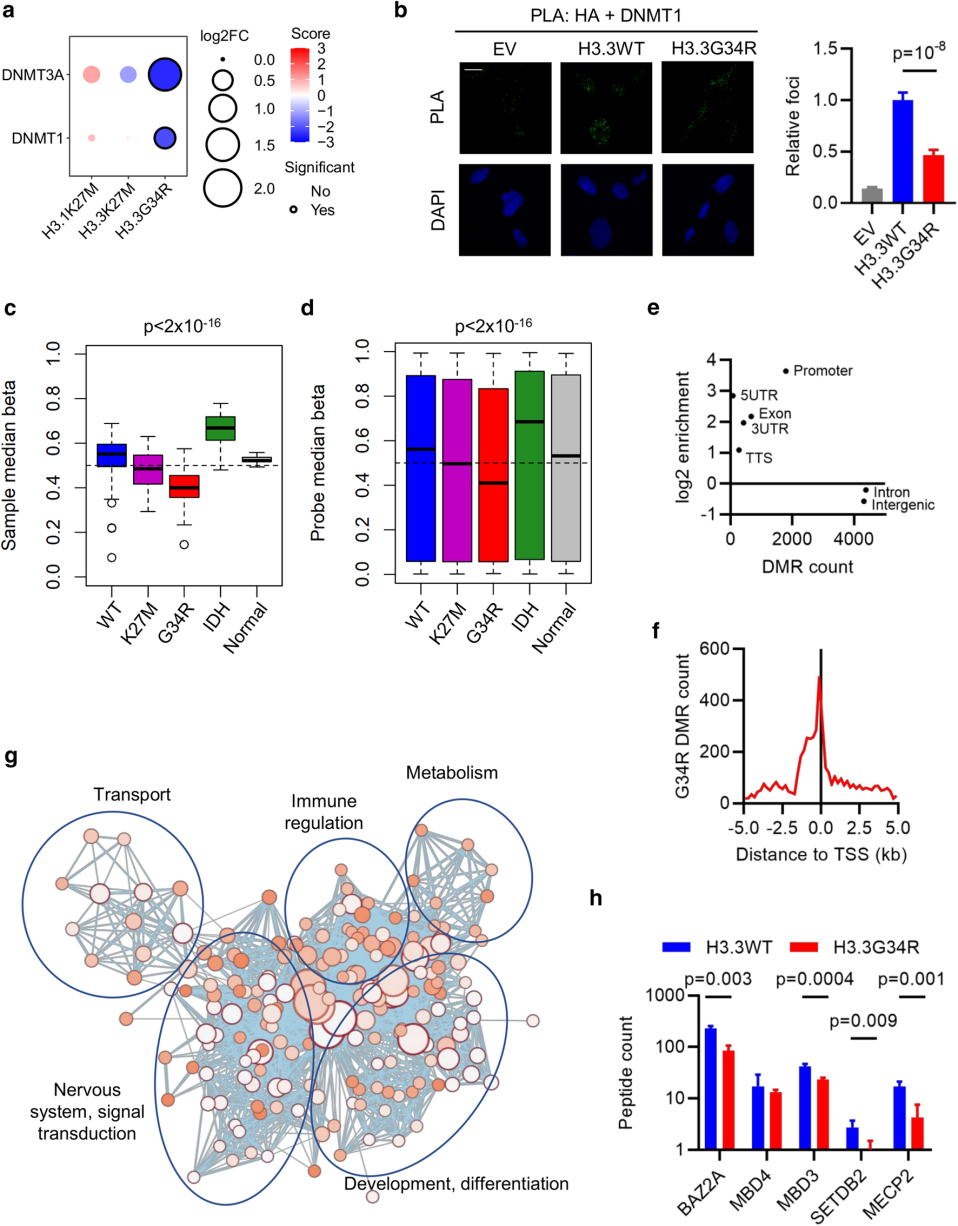
Reduced interaction with DNA methyltransferases leads to global hypomethylation in H3.3G34R-mutant pHGG. **A** Bubble plot showing differential enrichment of DNMT1 and DNMT3A with each oncohistone relative to WT control. Size = log2(mutant/WT). Color is a function of -log10 p-value and direction of interaction change. Statistically significant (*p* < 0.05) differential interactions have a black border. **B** Proximity ligation assays in MO3.13 cells between HA-H3 and DNMT1. Scale bar: 20 μm. Results are representative of two biological replicates and show mean foci counts relative to H3.3WT ± standard error. *n*: EV = 52; H3.3WT = 73; H3.3G34R = 61. *p*: ANOVA. **C** Median methylation beta value per sample from pHGG categorized as WT (*n* = 98); mutant for K27M (n = 34), G34R (*n* = 24), or IDH (*n* = 25); or normal brain (*n* = 7). The box marks the interquartile range (IQR) and shows the median value. The whiskers extend to 1.5 × IQR. *p*: ANOVA. **D** Median methylation beta value per probe from pHGG categorized as WT (*n* = 98); mutant for K27M (*n* = 34), G34R (*n* = 24), or IDH (*n* = 25); or normal brain (*n* = 7). The box marks the interquartile range (IQR) and shows the median value. The whiskers extend to 1.5 × IQR. *p*: ANOVA. **E** Scatter plot of number of differentially methylated regions (DMR) detected in H3.3G34R-mutant (*n* = 24) vs WT (*n* = 98) tumors assigned to each genomic location category versus the log2-enrichment against an expected background. **F** Enrichment of DMRs detected in H3.3G34R-mutant (*n* = 24) vs WT (*n* = 98) tumors around transcription start sites (TSS). **G** Network of pathways (Reactome, KEGG, Gene Ontology Biological Processes) enriched in genes with DMRs ± 1 kb from the TSS. Node size and color reflects significance and edge thickness reflects number of genes shared between two nodes. **H** Peptide counts of methyl-binding domain (MBD) containing proteins identified by BioID with H3.3WT or H3.3G34R. *p*: *t* test

H3K27M mutations lead to global DNA hypomethylation [10, 16], and we expected to see this reflected by a loss of interaction with DNA methyltransferases (DNMT). Surprisingly, neither H3.1K27M or H3.3K27M had significantly altered DNMT interaction, while H3.3G34R lost association with both DNMT1 and DNMT3A (Figs. 1f, 3a). PLA confirmed the loss of binding of H3.3G34R, compared with H3.3WT, to DNMT1 in ectopic H3-expressing MO3.13 cells (*p* < 0.0001; Fig. 3b). This suggests that H3K27M affects DNA methylation through a mechanism other than DNMT binding, and that H3.3G34R mutations also affect DNA methylation. To assess this possibility, we examined DNA methylation in a large panel of pHGG that were H3K27M-, H3.3G34R-, or IDH-mutant with tumors that were WT for each of these drivers and normal brain controls [10, 27]. The groups were significantly different from one another *(p* < 2 × 10^–16^, ANOVA), with H3.3G34R-mutant tumors having the lowest genome-wide methylation states at both sample and individual probe levels (Fig. 3c, d).

Among 13,628 differentially methylated regions (DMRs) between H3.3G34R and WT tumors, 88% had reduced methylation in H3.3G34R-mutant tumors. H3.3G34R-associated DMRs were enriched in promoters and transcription start sites (TSS) (Fig. 3e, f), broadly distributed across the genome, and associated with 6234 genes. Genes with a DMR within ± 1 kb of the TSS function in metabolism, development and differentiation as well as nervous system signal transduction (Fig. 3g). Enrichment of developmental and neural pathways is consistent with findings that H3.3G34R mutations lead to altered differentiation [18]. Metabolic enrichment is consistent with our finding of metabolic protein binding and increased mitochondrial metabolism in H3.3G34R-mutant cells (Figs. 1e, 2a, e, f, g; Supp. Fig. S2a, online resource).

Finally, the H3.3G34R association of methyl-binding domain (MBD) containing proteins was reduced (Fig. 3h), suggesting that, as well as globally regulating DNA methylation, H3.3G34R directly affects local DNA methylation. Collectively, H3.3G34R-mutant tumors have a wide-spread and pronounced DNA hypomethylation phenotype that is more severe than H3.3K27M-driven tumors.

### H3 K27M and G34R mutants have opposite transcription factor association

**Fig. 4:**
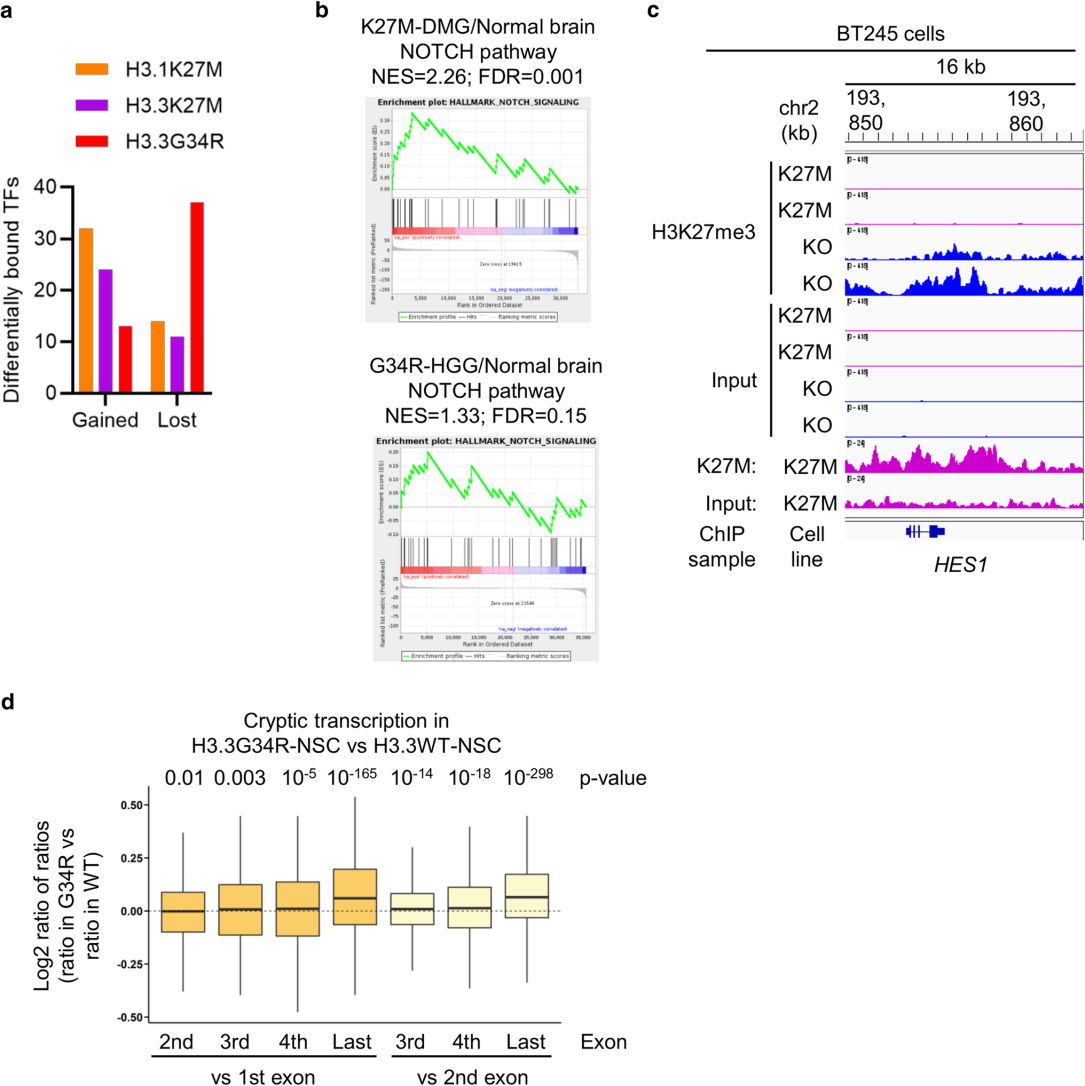
Oncohistones alter the transcription factor landscape. **A** Differentially bound TFs with each oncohistone relative to its WT control. **B** Gene set enrichment analysis (GSEA) of NOTCH pathway in H3K27M-mutant DMG (*n* = 38) compared with normal brain (*n* = 20), and H3.3G34R-mutant pHGG (*n* = 20) compared with normal brain (*n* = 5). *NES* normalized enrichment score. *FDR* false discovery rate. **C** H3K27me3 and K27M ChIP-Seq read density in a window centered on the *HES1* gene in BT245 cells that are either parental or modified with CRISPR to remove the H3.3K27M mutation. **D** Cryptic transcription levels in H3.3G34R (*n* = 2) vs H3.3WT (*n* = 2) NSCs. Exon-level expression levels were calculated and the ratio against first or second exon expressions were compared between cell lines. *p*: Wilcoxon rank sum test

Transcription-related pathways were enriched with H3.1K27M and H3.3K27M and reduced with H3.3G34R (Fig. 1e; Supp. Fig. S2a, online resource). Deposition into different promoter regions and/or local chromatin regulation will alter oncohistone exposure to transcription factors (TFs) regulating gene expression, as well as have the potential to influence TF recruitment to those promoters. Comparison with known human TFs [53] revealed that all 3 mutants both gained and lost TF associations, however, while both K27M mutants gained ~ 2 × more TF associations than they lost, H3.3G34R lost ~ 3 × more TFs than it gained (Fig. 4a; Supp. Fig. S4a-c, online resource). Five TFs (ATF1, DLX6, DRAP1, GATA4, HOXC9) gained interaction with all 3 mutants, while 2 TFs (ADNP2, SPEN) had reduced interaction with all 3 (Supp. Fig. S4b-d, online resource).

Although many TFs have cell-type specific expression and effects, we were intrigued by the reduced binding to Spen family transcriptional repressor (SPEN), a NOTCH repressor, by all 3 oncohistones, which would be expected to lead to NOTCH activation. In H3K27M-mutant diffuse midline glioma (DMG) and H3.3G34R-mutant pHGG, the NOTCH pathway was upregulated (Fig. 4b). Removal of the H3.3K27M mutation by CRISPR-mediated genome editing in the H3.3K27M-mutant BT245 cell line [35] resulted in restoration of high-levels of H3K27me3 across the gene body of *HES1*, a NOTCH effector TF (Fig. 4c). NOTCH pathway gene expression has previously been reported to be activated by both H3.3K27M and H3.3G34R [19, 28]. Together, this suggests that oncohistones activate NOTCH signaling in pHGG at least in part via loss of association with SPEN, coupled with epigenetic modulation of NOTCH target genes.

The gain of TF association with both H3.1K27M and H3.3K27M is consistent with genome-wide H3K27me3 loss creating large genomic regions with increased TF accessibility, which are more permissive for transcriptional remodeling. In contrast, the mechanism with H3.3G34R is not as clear. Local H3K36me3 reduction in genes with H3.3G34R deposition would not be expected to result in increased TF access, in agreement with our data and findings that H3.3G34R has relatively modest transcriptomic effects [19, 28]. Instead, it would be anticipated to limit access by HDACs, DNMTs and DNA repair factors (Fig. 1e, f; Supp. Fig. S2, online resource).

One effect of H3K36me3 deposition within gene bodies is to suppress cryptic transcription initiation from internal start sites, a function that is mediated by recruitment of SETD2 and DNMTS in conjunction with RNA polymerase. We therefore hypothesized that H3.3G34R and its consequently reduced H3K36me3 and DNA methylation would increase cryptic transcription.

To test this hypothesis, we re-analyzed RNA-Seq data from human fetal NSCs with or without H3.3G34R expression [13]. Cryptic initiation of transcription within a gene increases coverage in downstream exons relative to earlier exons. We therefore measured exon-level expression of multi-exon genes and compared the expression in transcripts per million (TPM) of later exons to the first or second exon; comparison of these ratios between samples allows the relative amount of cryptic transcription to be assayed with increased ratios of later vs earlier exons suggesting more cryptic transcription [63]. In H3.3G34R-mutant cells, the exon-TPM ratio increased compared to H3WT, consistent with an increase in cryptic transcription downstream from H3.3G34R (Fig. 4d). The effect was more pronounced in more highly expressed genes (Supp. Fig. S4e, online resource). Suppression of cryptic transcription maintains transcriptional fidelity by preventing initiation of spurious transcripts and re-establishing a repressive chromatin state [66]. Cryptic transcription occurs in aging cells [63] as well as in cancer [22] where it may serve to increase tumor immunogenicity and susceptibility to immunotherapy [15].

### Both K27M and G34R alter H3K9 modifier interactions

H3K9 methyltransferases had reduced interaction with all 3 oncohistones, while H3.3G34R additionally lost H3K9 demethylase interactions (Fig. 1e, 5a). SUV39H1, SUV39H2 and SETDB2 (as well as SETDB1, which was not recovered by BioID), write H3K9me2/3, while EHMT1/2 and PRDM2 write H3K9me1/2 [42]. H3.3K27M had reduced association with trimethylases while H3.1K27M and H3.3G34R also had reduced association with mono/di-methylases. H3.3G34R lost interactions with KDM3 and KDM7 (PHF2/8) family members, which demethylate H3K9me1/2, as well as KDM4B, which demethylates H3K9me3 [42]. This would be expected to lead to reduced H3K9 methylation in association with H3K27M, and harder to predict consequences for H3.3G34R.

**Fig. 5:**
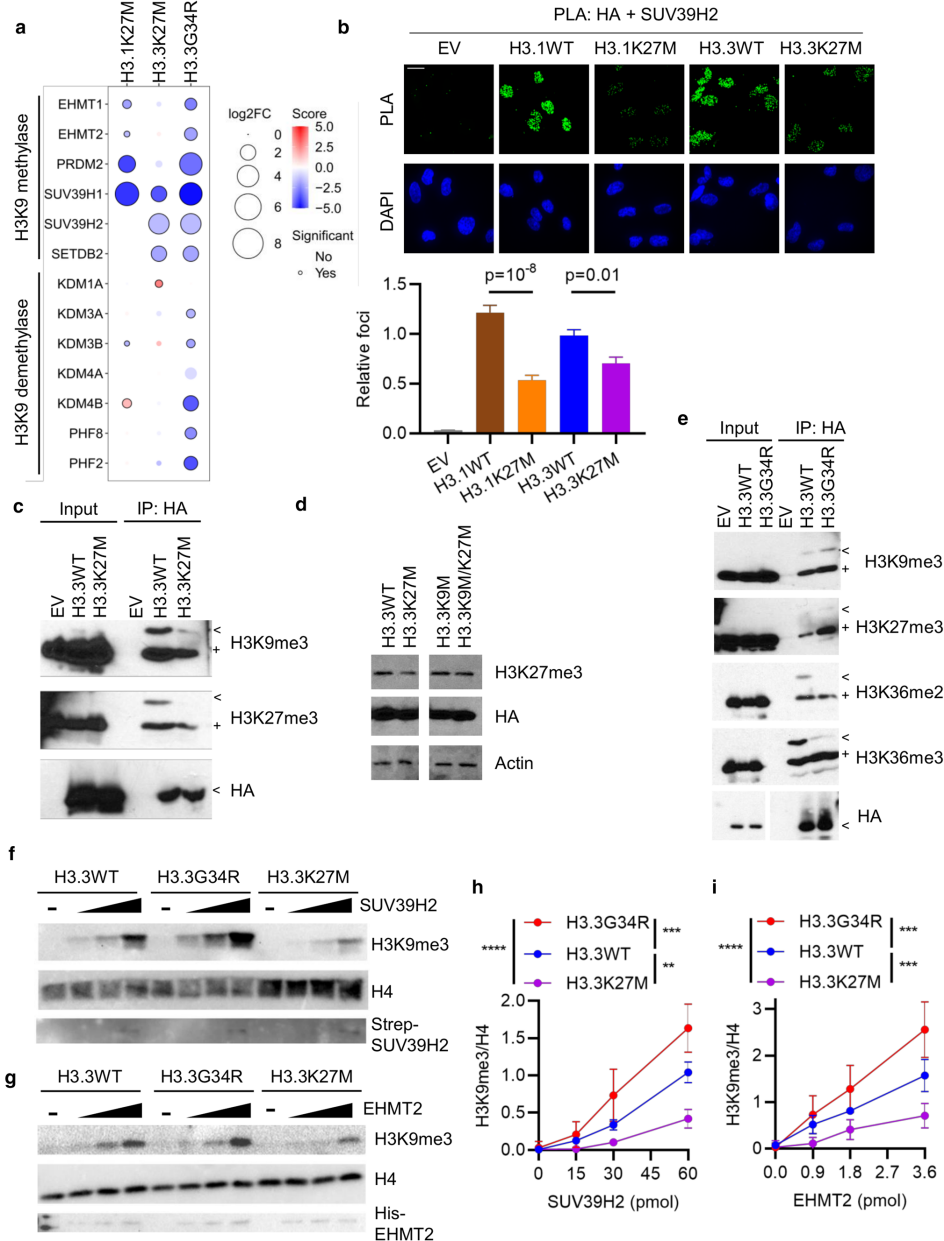
Oncohistones disrupt H3K9 methylation. **A** Bubble plot showing differential enrichment of indicated H3K9 methylases and demethylases with each oncohistone relative to WT control. Size = log2(mutant/WT). Color represents significance. Statistically significant (*p* < 0.05) differential interactions have a black border. **B** Proximity ligation assays in MO3.13 cells between HA-H3 and SUV39H2. Scale bar: 20 μm. Results are representative of two biological replicates and show mean foci counts relative to H3.3WT ± standard error. *n*: EV = 14; H3.1WT = 36; H3.1K27M = 67; H3.3WT = 60; H3.3K27M = 62. *p*: ANOVA. **C** Ectopic histone-containing nucleosomes were immunoprecipitated (IP) from H3-expressing NHA cells and ectopic and endogenous modifications were assessed by western blotting. < : Ectopic HA-tagged histone. + : endogenous histone. **D** NHA cells were transduced with indicated constructs and whole cell lysates analyzed by western blotting. **E** Ectopic histone-containing nucleosomes were immunoprecipitated (IP) from H3-expressing NHA cells and ectopic and endogenous modifications were assessed by western blotting. < : Ectopic HA-tagged histone. + : endogenous histone. **F** Representative gels from in vitro methylation assays carried out with nucleosomes assembled with H3.3WT, H3.3G34R or H3.3K27M and incubated with increasing amounts of SUV39H2. **G** Representative gels from in vitro methylation assays carried out with nucleosomes assembled with H3.3WT, H3.3G34R or H3.3K27M and incubated with increasing amounts of EHMT2. **H** Quantification of SUV39H2 methylation assays from **F** (*n* = 3). *p* (ANOVA): H3.3WT vs H3.3K27M, *p* = 0.003; H3.3WT vs H3.3G34R, *p* = 0.001; H3.3K27M vs H3.3G34R, *p* = 10^–7^. **I** Quantification of EHMT2 methylation assays from **G** (*n* = 3). *p* (ANOVA): H3.3WT vs H3.3K27M, *p* = 0.006; H3.3WT vs H3.3G34R, *p* = 0.009; H3.3K27M vs H3.3G34R, *p* = 10^–6^

Analysis of RNA-Seq data [18, 69, 78] showed that, overall, the expression levels of the H3K9 methyltransferases and demethylases in H3-mutant pHGG is not drastically altered compared with normal brain (Supp. Fig. S5a, online resource). *PRDM2* was the only differentially expressed gene in either H3K27M or H3.3G34R mutant pHGG (log2-fold change H3K27M versus normal brain = − 1.16, *p* = 0.0007). This suggests these genes are not transcriptionally regulated to compensate for any reduced activity at the protein level and that altered H3K9 modification downstream from oncohistones could potentially be exploited therapeutically.

We next confirmed the reduced interaction of H3.3K27M and H3.1K27M with SUV39H1 and SUV39H2 with PLA in mutant or WT H3-expressing MO3.13 cells along with EHMT2, which had a reduced interaction with H3.1K27M but not H3.3K27M, consistent with the BioID data (Fig. 5b; Supp. Fig. S5b, d, online resource). In contrast to BioID where H3.3G34R lost interaction with SUV39H2 and EHMT2, PLA showed a gain of association (Supp. Fig. S5c, e, online resource). This discrepancy could have arisen for a variety of reasons, including the interaction radius of BioID (10 nm) [51] being smaller than for PLA (40 nm) [1], which could allow PLA to capture indirect associations with further-afield nucleosomes.

To test the functional consequences of the oncohistones on enzyme activity we used NHAs expressing WT or mutant histone to profile histone modifications. Western blotting showed that, as expected, both H3.1K27M and H3.3K27M globally reduced H3K27me3 while H3.3G34R did not affect global H3K36me3; similarly, total H3K4me3 and H3K9me3 was unchanged (Supp. Fig. S5f, online resource). Consistent with recent data, H3.1K27M had a larger effect on H3K27me3 than H3.3K27M [74]. While bulk H3K9me3 was unaffected, it was possible that they may have localized effects. We therefore immunoprecipitated exogenous histones from dinucleosomes isolated from NHAs expressing H3.3WT, H3.3K27M or H3.3G34R. H3K9me3 was reduced on both the exogenous and endogenous histones of H3.3K27M-containing dinucleosomes (Fig. 5c), in agreement with BioID showing reduced association with SUV39H1/2 and SETDB2 (Fig. 5a). To further explore the consequence of H3K9 methylation in the context of H3K27M, we expressed H3.3K27M and H3.3K9M, either separately or in the same H3 construct, in HEK293T cells. While H3.3K9M on its own did not affect H3K27me3, the H3.3K9M/K27M double mutant blocked global reduction of H3K27me3, underscoring the importance of H3K9 methylation for the dominant negative effect of H3K27M, and suggesting H3K9 methyltransferases as a potential therapeutic target (Fig. 5d). In H3.3G34R-mutant nucleosomes, H3K36me2/3 was lost on H3.3G34R but not its WT H3 partner. This was accompanied by an increase in H3K27me3 on both ectopic H3.3G34R and endogenous H3 (Fig. 5e), consistent with previous findings [13, 18, 44, 48, 56]. H3.3G34R-K9me3 was increased compared with H3.3WT, and there was also a slight increase in H3K9me3 on WT H3 bound to H3.3G34R (Fig. 5e).

The altered H3K9me3 on oncohistones could be a direct effect on methyltransferases, or result from steric hindrance by PRC2 or another complex tightly bound to the mutant. To explore this, we conducted in vitro methylation assays with nucleosomes reconstituted with WT or mutant H3.3 and purified SUV39H2 and EHMT2. Increasing amounts of enzyme titrated into the reaction mixture led to a concentration-dependent increase of H3K9me3 on all 3 histones (Fig. 5f–i). SUV39H2 and EHMT2 were significantly less active towards H3.3K27M (*p* ≤ 0.01), and significantly more active towards H3.3G34R (*p* < 0.001). This striking result suggests that distal mutant residues on the histone tail differently affect the activity of these enzymes.

Collectively, these data support a model whereby the broad epigenetic changes induced by H3K27M are exacerbated by a local loss of H3K9 methyltransferase activity and H3K9me3. In contrast, H3.3G34R mutations lead to a local increase in H3K9me3 through increased H3K9 methyltransferase binding and kinetics as well as altered H3K9 demethylase binding.

### H3K9me3 is a therapeutic vulnerability in histone-mutant pHGG

**Fig. 6:**
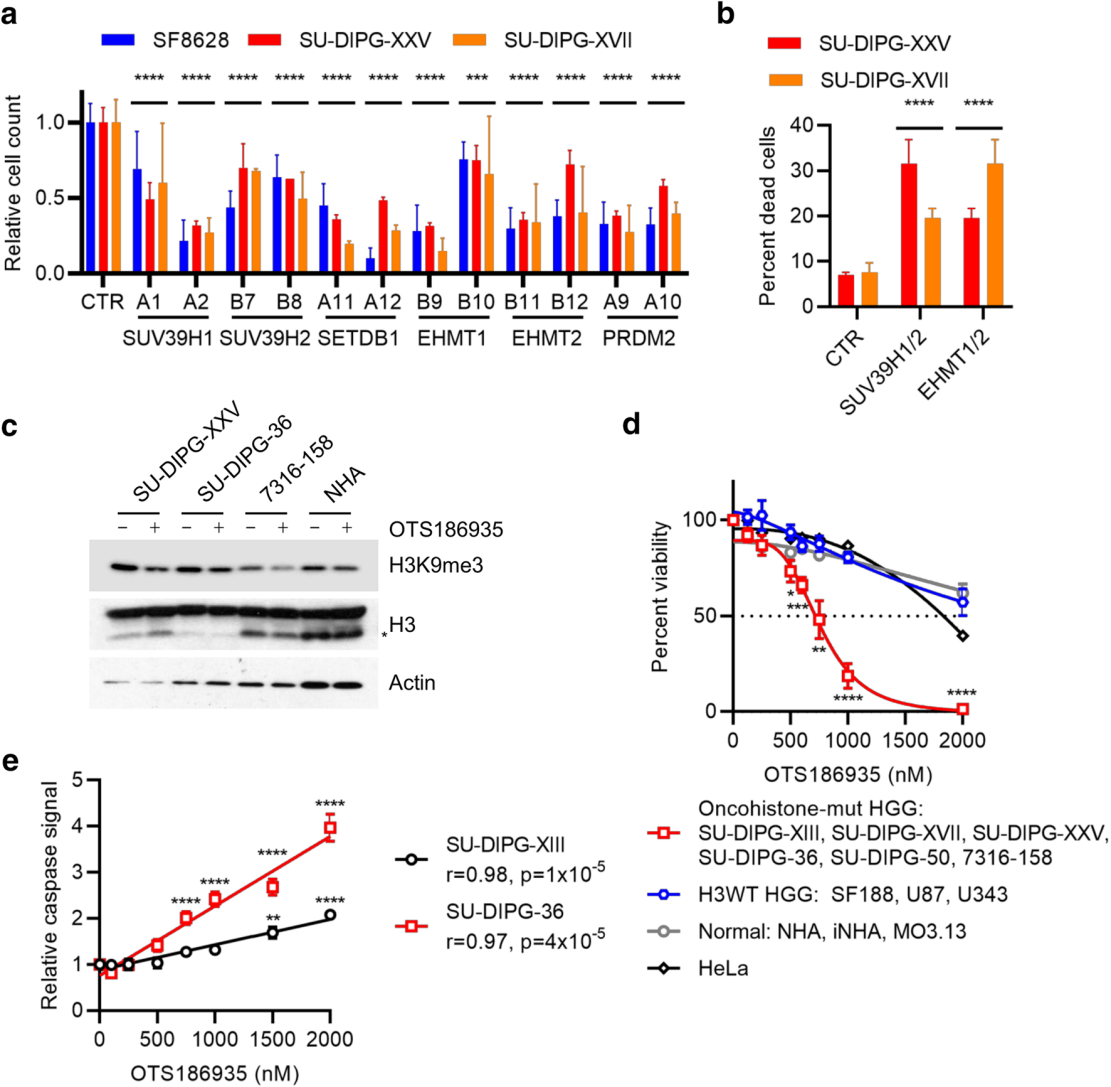
Oncohistone-mutant pHGG cells are vulnerable to inhibition of H3K9 methylation. **A** Viable cell counts of H3.3K27M-mutant cell lines transduced with shRNA lentiviral constructs targeting H3K9 methylases, expressed relative to cells transduced with control shRNA. Two separate shRNA clones were used for each gene. Results show mean ± standard deviation of 2–5 biological replicates. *p*: ANOVA comparing each clone to control shRNA (CTR). ****p < 0.0001. **B** Percent cell death of H3.3K27M-mutant cell lines transduced with combinations of control shRNA targeting H3K9 methylases. Results show mean ± standard deviation of 3 biological replicates. *p*: ANOVA comparing each clone to control shRNA (CTR). ****p < 0.0001. **C** Cells treated with DMSO or OTS186935 (1,500 nM) for 4 days were analyzed by western blotting. *: clipped H3. **D** Relative viable cell counts of cell lines treated for 4 days with DMSO or increasing doses of OTS186935. Results show mean ± standard error for groups of cell lines. Each cell line was analyzed in six biological replicates. **E** Quantification of caspase activation assay in SU-DIPG-XIII and SU-DIPG-36 cells treated with DMSO or increasing doses of OTS186935 and measured after 24 h. Results show mean ± standard error of 6 biological replicates, with 2 fields of view measured per replicate. **p < 0.01; ****p < 0.0001; *r*: Pearson correlation. *p* values for overall correlation were determined with a *t* test and for comparisons to DMSO by ANOVA with Dunnett’s multiple comparisons test

Having established that both H3G34R and H3K27M affect local H3K9me3 and that H3K9 modification status is important for the global effect of H3K27M on H3K27me3, we explored whether this could be exploited therapeutically. H3.3K27M-mutant pHGG cell lines (SF8628, SU-DIPG-XXV, SU-DIPG-XVII) were transduced with shRNA targeting H3K9 methyltransferases identified by BioID (H3K9me3: SUV39H1, SUV39H2, SETDB2; H3K9me1/2: EHMT1, EHMT2, PRDM2). All led to a significant reduction in cell viability compared with control shRNA (*p* < 0.0001; Fig. 6a). For the non-adherent SU-DIPG-XXV line, both the number and size of the spheres were reduced (Supp. Fig. S6a, online resource). In H3.3G34R-mutant cells (7316-158), we found a similar sensitivity to shRNA targeting SUV39H1, SUV39H2, EHMT1 and EHMT2 as we observed for H3.3K27M-mutant cells (Supp. Fig. S6b, online resource), suggesting that altered the altered balance of local H3K9 methylation in oncohistone mutant cells is important for their viability.

EHMT1 and EHMT2 function as homo- or heterodimers, while SUV39H1 and SUV39H2 are partially redundant [42]. Therefore, knockdown of one enzyme alone could be at least partly compensated for by its partner. SU-DIPG-XXV and SU-DIPG-XVII cells were more sensitive to simultaneous depletion of EHMT1/2 than to single knockdowns, and SU-DIPG-XXV were also more sensitive to SUV39H1/2 double knockdown (Supp. Fig. S6c, online resource). The double knockdowns induced cell death, indicating that the H3K9 methyltransferase loss is cytotoxic rather than cytostatic (Fig. 6b).

In parallel, we also tested selective H3K9 methyltransferase inhibitors. We first treated a panel of cell lines, including normal, H3K27M and H3G34R mutant lines, with OTS186935, a specific inhibitor of SUV39H2 [82]. As expected, H3K9me3 was reduced in all cells following treatment for 5 days (Fig. 6c). Cell viability assays showed that oncohistone-mutant (H3.3K27M: SF8628, SU-DIPG-XXV, SU-DIPG-XVII; H3.1K27M: SU-DIPG-36; H3.3G34R: 7316-158) cell lines had significant sensitivity to OTS186935, with an average IC50 of 763 nM (Fig. 6d; Supp. Fig. S6d, online resource). In sharp contrast, the IC50 values for control cell lines were significantly higher for normal (NHA, iNHA, MO3.13: IC50 3,082 nM), HeLa (IC50 ~ 1,800 nM) and H3WT glioma (SF188, U87, U343: IC50 2,199 nM) cells (Fig. 6d; Supp. Fig. S6d, online resource). Caspase activation assays showed a dose dependent response to 24 h’ treatment with OTS186935 (*p* = 10^–5^; Fig. 6e), with significant increases in apoptosis with doses > 750 nM (*p* < 0.0001, ANOVA) in SU-DIPG-36 and > 1,500 nM (*p* ≤ 0.01, ANOVA) in SU-DIPG-XIII (Fig. 6e), consistent with the cell death observed in shRNA-transduced cells (Fig. 6b).

Next, we treated cells with chaetocin, which potently inhibits SUV39H1/2 and, to a lesser extent, EHMT1/2 [32]. After 24 h, H3K9me3 was reduced alongside γ-H2AX induction (Supp. Fig. S6e, online resource). Oncohistone mutant primary patient lines (H3.3K27M: SF8628, SU-DIPG-XXV, SU-DIPG-XVII; H3.1K27M: SU-DIPG-36; H3.3G34R: 7316-158) were exquisitely sensitive to chaetocin, with 125 nM sufficient to reduce the viable cell count by at least half compared with DMSO, with robust induction of cell death (Supp. Fig. S6f-g, online resource). Caspase activation assays showed apoptosis in SU-DIPG-XVII and SU-DIPG-50 cells after 24 h’ chaetocin treatment (Supp. Fig. S6h, online resource). Overall, these data support a model in which oncohistone-mutant pHGG cells are vulnerable to altered H3K9 methylation state and suggest a novel therapeutic opportunity.

## Discussion

Using BioID to characterize the interactome of 3 oncogenic driver histone mutations (H3.1K27M, H3.3K27M and H3.3G34R) we identified key differences in their oncogenic mechanisms as well as common therapeutic vulnerabilities. All undergo broad changes in their protein associations, with our data implicating a predominantly epigenetic/chromatin regulation-based mechanism with increased availability of DNA for transcription factors for H3K27M. H3.3G34R was associated with reduced transcription factor binding, DNA methylation, and DNA repair; and increased cryptic transcription and mitochondrial metabolism.

Our data confirmed the known increased association between H3K27M and the PRC2 core complex, which results in global H3K27me3 loss accompanied by increases in H3K36me2 [10, 56]. Intriguingly, we found that H3.3K27M has increased interaction with both PRC2.2 and PRC2.1 subcomplexes [36, 80] while H3.1K27M gains interaction only with PRC2.1. PRC2.1 and PRC2.2 have similar genome-wide profiles and are at least partly redundant in maintaining H3K27me3 [37, 39, 68, 88]. However, they are recruited to chromatin differently, and in pluripotent stem cells PRC2.1 has a higher chromatin affinity than PRC2.2 [88], suggesting they can also function independently and that the ratio of subcomplexes engaged with chromatin is also important. The different modes of chromatin deposition of H3.1 and H3.3 [30, 33], which are shared with their K27M mutants [65, 74] may account for these differences.

The increased binding of H3K27M to PRC2 and TFs supports a model whereby H3K27M’s effects are epigenetic/transcriptional. H3K27me3 removal across large genomic regions would allow activation of both PRC2 and non-PRC2 target genes [69] through increased TF accessibility. In contrast, H3.3G34R overall lost association with transcription factors.

H3K27M had unchanged DNMT interactions, which was surprising given the DNA hypomethylation in H3K27M-mutant tumors [10, 16]. However, EZH2 plays a role in DNMT recruitment [75, 81], and it is possible that altered H3K27M-mediated alterations in EZH2 function could mediate the global DNA hypomethylation. Meanwhile, H3.3G34R lost interaction with DNMT1 and DNMT3A. H3.3G34R-mutant tumors had stronger global DNA hypomethylation than H3K27M tumors, consistent with reduced interactions with both maintenance and de novo DNMTs. DNMT3A interacts with H3K36me3 through its PWWP domain, suggesting that the loss of this mark on H3G34R-mutant histones drives the DNA hypomethylation [8, 24].

H3.3G34R leads to local H3K36me2/3 loss and reduced binding to the H3K36me3 reader ZMYND11 [13, 44, 83]. H3.3G34R has also been shown to have altered association with spliceosome components, resulting in disrupted alternative splicing [58]. Localized H3K36me2/3 losses on H3.3G34R-mutant histones would not be expected to increase TF accessibility. Although H3K36me3 is deposited at promoters, a large part of its role is in gene bodies. Together with DNA methylation and histone deacetylation, its deposition in gene bodies reimposes a repressive chromatin environment in the wake of RNA polymerase-II passage. As well as affecting H3K36me3, H3.3G34R also had reduced interactions with DNMTs and HDAC. This suggested that, unlike H3K27M, where broad loss of H3K27me3 leads to increased transcription, H3.3G34R could misregulate transcription by other means including by perturbing DNMT/HDAC/SETD2-mediated repressive chromatin within gene bodies, which would result in increased cryptic transcription. Indeed, our data show increased cryptic transcription in H3.3G34R mutant cells compared with their WT counterparts. Cryptic transcription has previously been reported in cancer, where it may increase tumor immunogenicity, and could represent an Achilles heel for these tumors [15, 16, 22].

The oncohistone chromatin environment was extensively remodeled as indicated by altered chromatin regulator associations. Notably, H3K9 modifiers showed family-wide changes in oncohistone binding. H3K27M-containing nucleosomes had reduced H3K9me3, mediated by reduced H3K9 methylase association. An H3K9M/K27M double mutant abrogated the genome-wide H3K27me3 loss induced by H3K27M, suggesting that the remaining H3K9me3 on H3K27M histones may be important for the oncogenic effects of this mutation. However, K-M substitutions in the histone tail have increased SET domain binding (H3K4M, MLL; H3K27M, PRC2; H3K36M, NSD2) [45, 56, 60], so the double mutant could also have higher affinity for H3K9 methylases than PRC2.

H3.3G34R had altered binding to H3K9 methylases and demethylases. The methylase-demethylase balance determines H3K9me3 levels. H3.3G34R-containing nucleosomes had increased H3K9me3, suggesting that the loss of demethylases outweighs the loss of methylases.

H3.1K27M, H3.3K27M or H3.3G34R mutant pHGG cells were sensitive to H3K9 methylase disruption. Inhibitors and shRNA induced apoptotic cell death, suggesting a dependence on H3K9me3. H3K27me3 and H3K9me3 are both repressive modifications that can be mutually exclusive or cooperative, depending on context [90]. Disruption of the H3K27me3/H3K36me3 equilibrium by H3K27M and H3.3G34R may lead to compensatory H3K9me3 usage by tumor cells, leaving them vulnerable to its loss and providing an attractive therapeutic option for targeting these truncal mutations.

Beyond immediate transcription- and chromatin-related effects, we also observed that H3.3G34R-mutant cells have altered mitochondrial metabolism and that H3.3G34R itself interacts with mitochondrial proteins, with a fraction localized to the mitochondria. Among the effects of H3.3G34R, we found an increase in TCA cycle metabolites, similar to recent observations with H3K27M, where high α-ketoglutarate production helps to maintain the global loss of H3K27me3 [20]. It is intriguing that, as with DNA methylation, both oncohistones target some of the same metabolic pathways, albeit with different mechanisms and effects. Intriguingly, we found that H3.3G34R cells had elevated fumarate and, to a lesser extent, succinate. These metabolites are known to inhibit α-ketoglutarate-dependent histone lysine demethylases [87], including the H3K9 demethylase KDM4 that has reduced interaction with H3.3G34R. This would be expected to further reduce the activity of KDMs towards H3.3G34R.

In summary, targeting mutant histones or their direct effectors has proven elusive to date. Here we show that oncohistones drive extensive remodeling of the histone-associated protein landscape. They disrupt chromatin homeostasis beyond their immediate, well characterized effects on H3K27me3/H3K36me3, including via H3K9me3. They disrupt transcription by diverse means including co-option of oncogenic signaling pathways through altered TF association and deregulating cryptic transcription; and deregulate metabolic processes. Overall, our data provides a strong rationale for therapeutically targeting these downstream effectors in oncohistone-mutant pHGG.

## Supplementary Information

Below is the link to the electronic supplementary material.

Supplementary file1 (PDF 1279 KB)

Supplementary file2 (XLSX 167 KB)

Supplementary file3 (XLSX 11 KB)

Supplementary file4 (XLSX 9 KB)
