# Supplementary material for: Oncohistone interactome profiling uncovers contrasting oncogenic mechanisms and identifies potential therapeutic targets in high grade glioma
Source: Acta Neuropathol. 2022 Sep 7;144(5):1027–48. doi: 10.1007/s00401-022-02489-2 (PMC9547787; doi:10.1007/s00401-022-02489-2)
Supplement: Supplementary file 1 — Supplementary file1 (PDF 1279 KB) [file 401_2022_2489_MOESM1_ESM.pdf]

**Figure S1. Histone BioID interactomes**

A. Heatmap depicting Pearson correlation of peptide counts associated with each BioID replicate.
B. Principal components analysis of all high-confidence interactors identified in the BioID experiment.
C. Percentage of oncohistone or WT variant interactors that are differentially bound.
D. Volcano plots of differential binding identified by BioID. Significantly gained or reduced interactors are highlighted ($|log2FC|>1$, $p<0.05$).
E. Proximity ligation assays in MO3.13 cells between HA-H3 and EZH2. Scale bar: 20 μm. Results of 3 biological replicates show mean fraction of the nucleus covered by interaction foci ± standard error. n: EV=133; H3.1WT=157; H3.1K27M=143; H3.3WT=123; H3.3K27M=113. p: ANOVA.
F. Proximity ligation assays in MO3.13 cells between HA-H3 and NSD1. Scale bar: 20 μm. Results of 2 biological replicates show foci counts relative to H3.3WT ± standard error. n: EV=156; H3.3WT=149; H3.3G34R=160. p: ANOVA.

**Figure S2. Oncohistone interactome enriched pathways**

A. Reactome and KEGG pathways significantly enriched (adjusted $p<0.05$) among proteins with significantly more or less binding to each oncohistone.
B. Bubble plot of significantly enriched (adjusted $p<0.05$) DNA damage-related Gene Ontology Biological Processes associated with gained and lost proteins for each oncohistone. Bubble size reflects the number of affected genes in each pathway, and the colour the -log10 adjusted p-value (red: gained terms, blue: lost terms).

**Figure S3. H3.3G34R localization in mitochondria**

A. Peptide counts from BioID of mitochondrial proteins with significant increase in binding to H3.3G34R (n=4).
B. Relative enrichment (log2(H3/control)) of mitochondrial outer membrane transporter (TOMM) proteins associated with each histone (n=4). p: t-test.
C. Relative enrichment (log2(H3/control)) of mitochondrial inner membrane transporter (TIMM) proteins associated with each histone (n=4). p: t-test.
D. Confocal microscopy of 7316-158 H3.3G34R-mutant pHGG cells stained with Mitotracker red and anti-H3.3G34R. Box shows zoom area at right. Arrows mark co-localization of H3.3G34R with mitochondria. Scale bar: 3 μm.
E. TFAM peptide counts detected by BioID (n=4). p: t-test.

**Figure S4. TF binding by oncohistones**

A. Heatmap of relative peptide counts of transcription factors identified by BioID. Average peptides per histone were Z-transformed.
B. Venn diagram comparing differentially gained interactors with each oncohistone with transcription factors.
C. Venn diagram comparing differentially lost interactors with each oncohistone with transcription factors.
D. Bubble plot showing differential enrichment of indicated transcription factors with each oncohistone relative to WT control. Size=log2(mutant/WT). Color is a function of -log10

p-value and direction of interaction change. Statistically significant ($p<0.05$) differential interactions have a black border.

E. Comparison of cryptic transcription levels in H3.3G34R mutant or WT NSCs (n=2). Genes were separated into quartiles. p: Wilcoxon rank sum test.

**Figure S5. Differential chromatin modifier binding by oncohistones**

A. RNA-Seq log2 fold-change versus normal brain of H3K27M (n=38; normal n=20) and H3.3G34R (n=20; normal n=5) pHGG. Bars show mean ± standard deviation.
B. Proximity ligation assays in MO3.13 cells between HA-H3 and SUV39H1. Scale bar: 20 μm. Results are representative of two biological replicates and show mean foci counts relative to H3.3WT ± standard error. n: H3.3WT=72; H3.3K27M=66. p: ANOVA.
C. Proximity ligation assays in MO3.13 cells between HA-H3 and SUV39H2. Scale bar: 20 μm. Results of two biological replicates show mean foci counts relative to H3.3WT ± standard error. n: EV=220; H3.3WT=203; H3.3G34R=199. p: ANOVA.
D. Proximity ligation assays in MO3.13 cells between HA-H3 and EHMT2. Scale bar: 20 μm. Results are representative of two biological replicates and show mean foci counts relative to H3.3WT ± standard error. n: EV=22; H3.1WT=50; H3.1K27M=60; H3.3WT=65; H3.3K27M=60. p: ANOVA.
E. Proximity ligation assays in MO3.13 cells between HA-H3 and EHMT2. Scale bar: 20 μm. Results are representative of two biological replicates and show mean foci counts relative to H3.3WT ± standard error. n: EV=279; H3.3WT=244; H3.3G34R=268. p: ANOVA.
F. Whole lysates from NHA cells transduced with indicated constructs were analyzed by Western blotting.

**Figure S6. Targeting H3K9 methylation in oncohistone mutant pHGG**

A. Brightfield images of SU-DIPG-XXV cells transduced for 4 days with shRNA.
B. Viable cell counts of 7316-158 cells (H3.3G34R-mutant) transduced with shRNA targeting H3K9 methylases, expressed relative to cells transduced with control shRNA. Results show mean ± standard deviation of 3 biological replicates. p: ANOVA relative to control shRNA (CTR). ****: $p<0.0001$.
C. Viable cell counts of H3.3K27M-mutant cell lines transduced for 4 days with combinations of shRNA targeting H3K9 methylases, expressed relative to cells transduced with control shRNA. Results show mean ± standard deviation of 3 biological replicates. p: ANOVA relative to control shRNA (CTR). ****: $p<0.0001$.
D. Heatmap of viability of cell lines treated for 4 days with increasing doses of OTS186935. Results are represented as the mean log10(percent viability relative to DMSO controls) of 6 biological replicates for each cell line.
E. SU-DIPG-XXV cells were treated for 24 hours with DMSO or chaetocin (125 nM) and whole cell lysates analysed by western blotting.
F. Relative viable cell counts of H3.3K27M-mutant cell lines treated for 4 days with DMSO or increasing doses of chaetocin. Results show mean ± standard deviation of 3 biological replicates. p: ANOVA comparing each concentration to DMSO. ****: $p<0.0001$.
G. Relative viable cell counts of pHGG cell lines treated for 4 days with DMSO or chaetocin (125 nM). Results show mean ± standard deviation of 3 biological replicates. p: t-test in each cell line relative to DMSO. ****: $p<0.0001$.

H. Percent cell death in H3.3K27M-mutant cell lines treated for 4 days with DMSO or increasing doses of chaetocin. Results show mean ± standard deviation of 3 biological replicates. p: ANOVA comparing each concentration to DMSO. ****: $p<0.0001$.

I. Quantification of caspase activation assay in in H3.3K27M-mutant cell lines treated with DMSO or chaetocin (125 nM) for 24 hours. Results show mean ± standard deviation of 3 biological replicates. p: ANOVA comparing each concentration to DMSO. ****: $p<0.0001$.

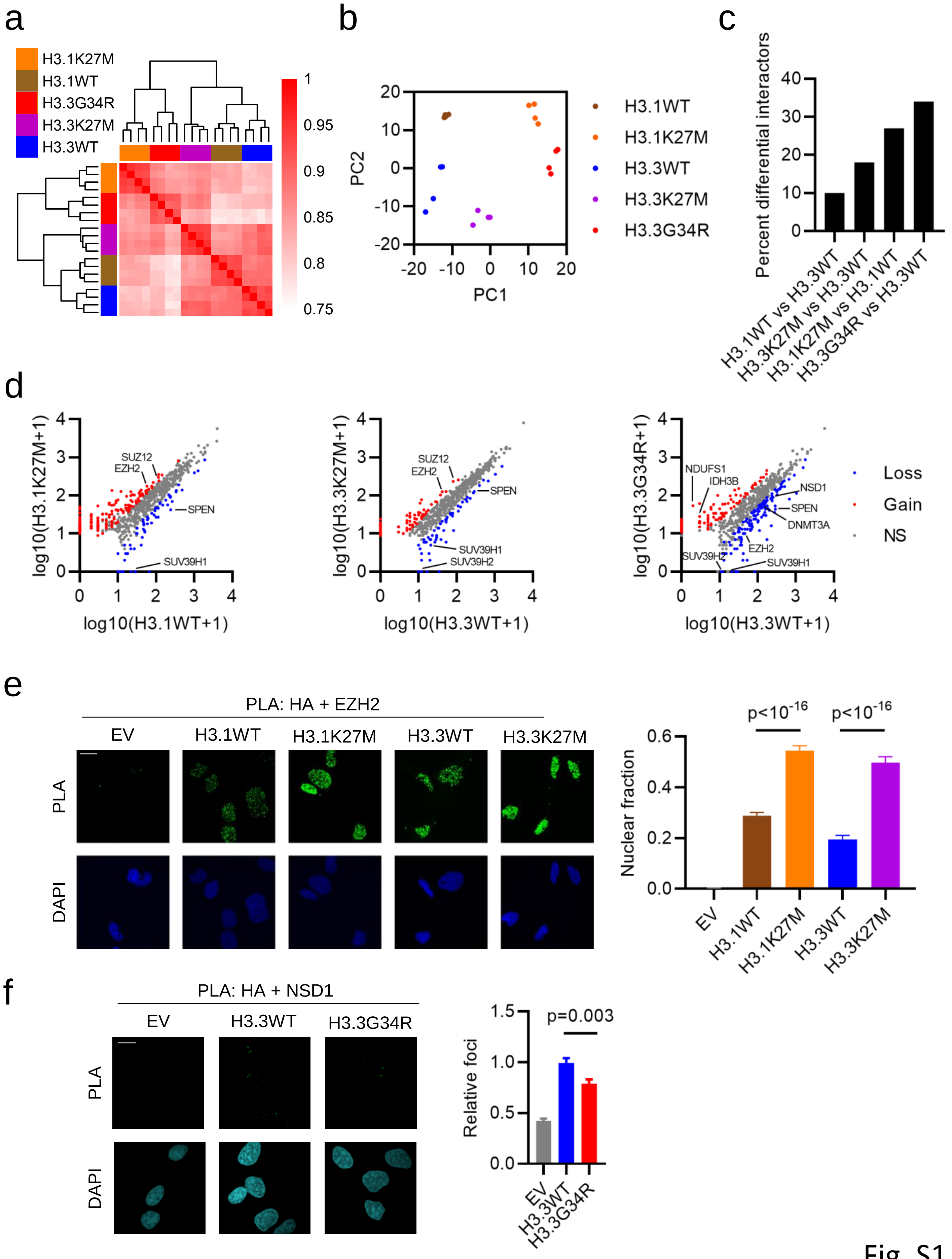
a
H3.1K27M
H3.1WT
H3.3G34R
H3.3K27M
H3.3WT
b
PC2
PC1
H3.1WT
H3.1K27M
H3.3WT
H3.3K27M
H3.3G34R
c
Percent differential interactors
H3.1WT vs H3.3WT
H3.3K27M vs H3.3WT
H3.1K27M vs H3.1WT
H3.3G34R vs H3.3WT
d
log10(H3.1K27M+1)
log10(H3.1WT+1)
SUZ12
EZH2
SPEN
SUV39H1
log10(H3.3K27M+1)
log10(H3.3WT+1)
SUV39H2
log10(H3.3G34R+1)
NDUFS1
IDH3B
NSD1
DNMT3A
Loss
Gain
NS
e
PLA: HA + EZH2
EV
H3.1WT
H3.1K27M
H3.3WT
H3.3K27M
PLA
DAPI
Nuclear fraction
p<10-16
f
PLA: HA + NSD1
H3.3G34R
Relative foci
p=0.003

Fig. S1

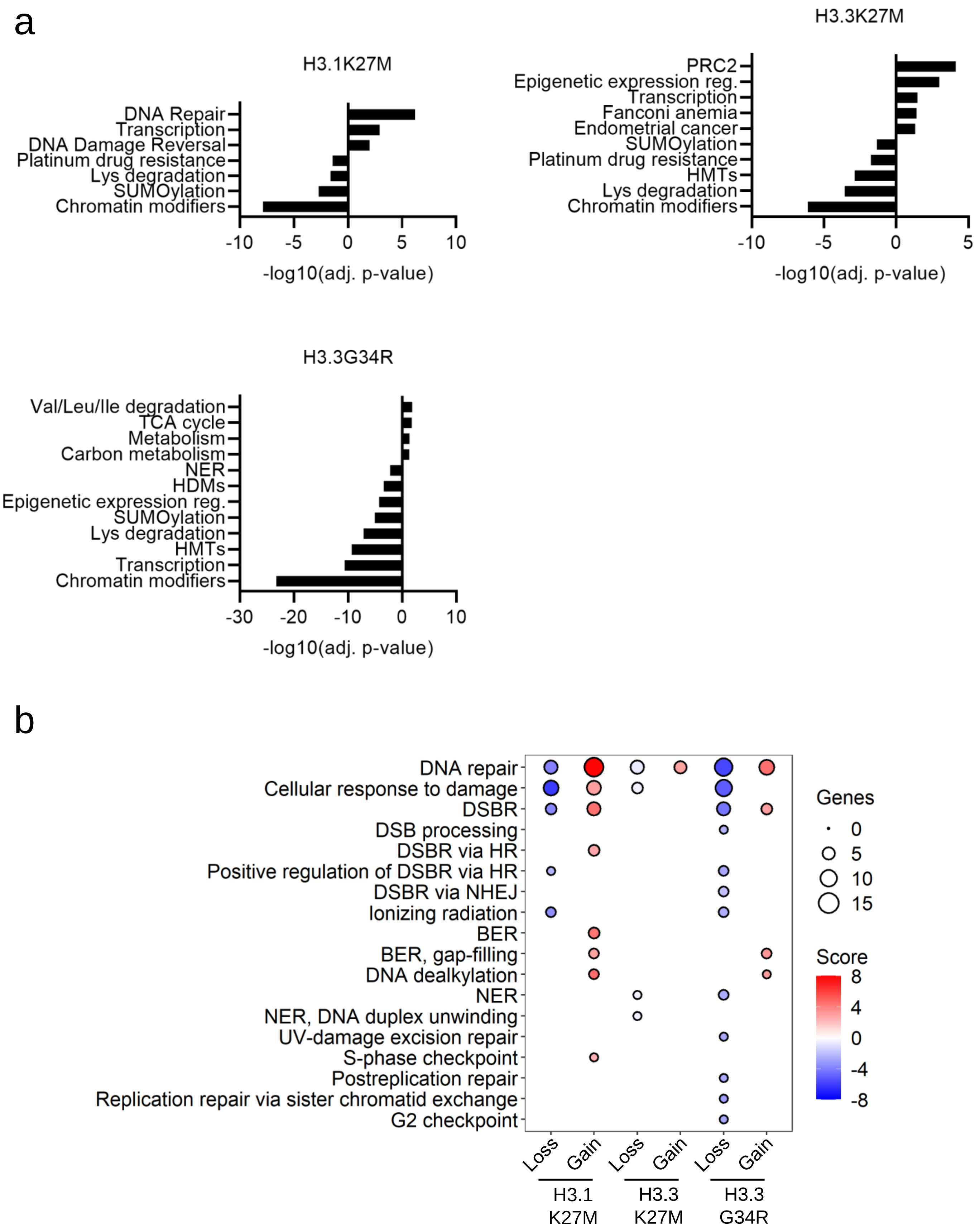
a
H3.1K27M
DNA Repair
Transcription
DNA Damage Reversal
Platinum drug resistance
Lys degradation
SUMOylation
Chromatin modifiers
-10
-5
0
5
10
-log10(adj. p-value)
H3.3K27M
PRC2
Epigenetic expression reg.
Transcription
Fanconi anemia
Endometrial cancer
SUMOylation
Platinum drug resistance
HMTs
Lys degradation
Chromatin modifiers
-10
-5
0
5
-log10(adj. p-value)
H3.3G34R
Val/Leu/Ile degradation
TCA cycle
Metabolism
Carbon metabolism
NER
HDMs
Epigenetic expression reg.
SUMOylation
Lys degradation
HMTs
Transcription
Chromatin modifiers
-30
-20
-10
0
10
-log10(adj. p-value)
b
DNA repair
Cellular response to damage
DSBR
DSB processing
DSBR via HR
Positive regulation of DSBR via HR
DSBR via NHEJ
Ionizing radiation
BER
BER, gap-filling
DNA dealkylation
NER
NER, DNA duplex unwinding
UV-damage excision repair
S-phase checkpoint
Postreplication repair
Replication repair via sister chromatid exchange
G2 checkpoint
Loss
Gain
Loss
Gain
Loss
Gain
H3.1 K27M
H3.3 K27M
H3.3 G34R
Genes
0
5
10
15
Score
8
4
0
-4
-8

Fig. S2

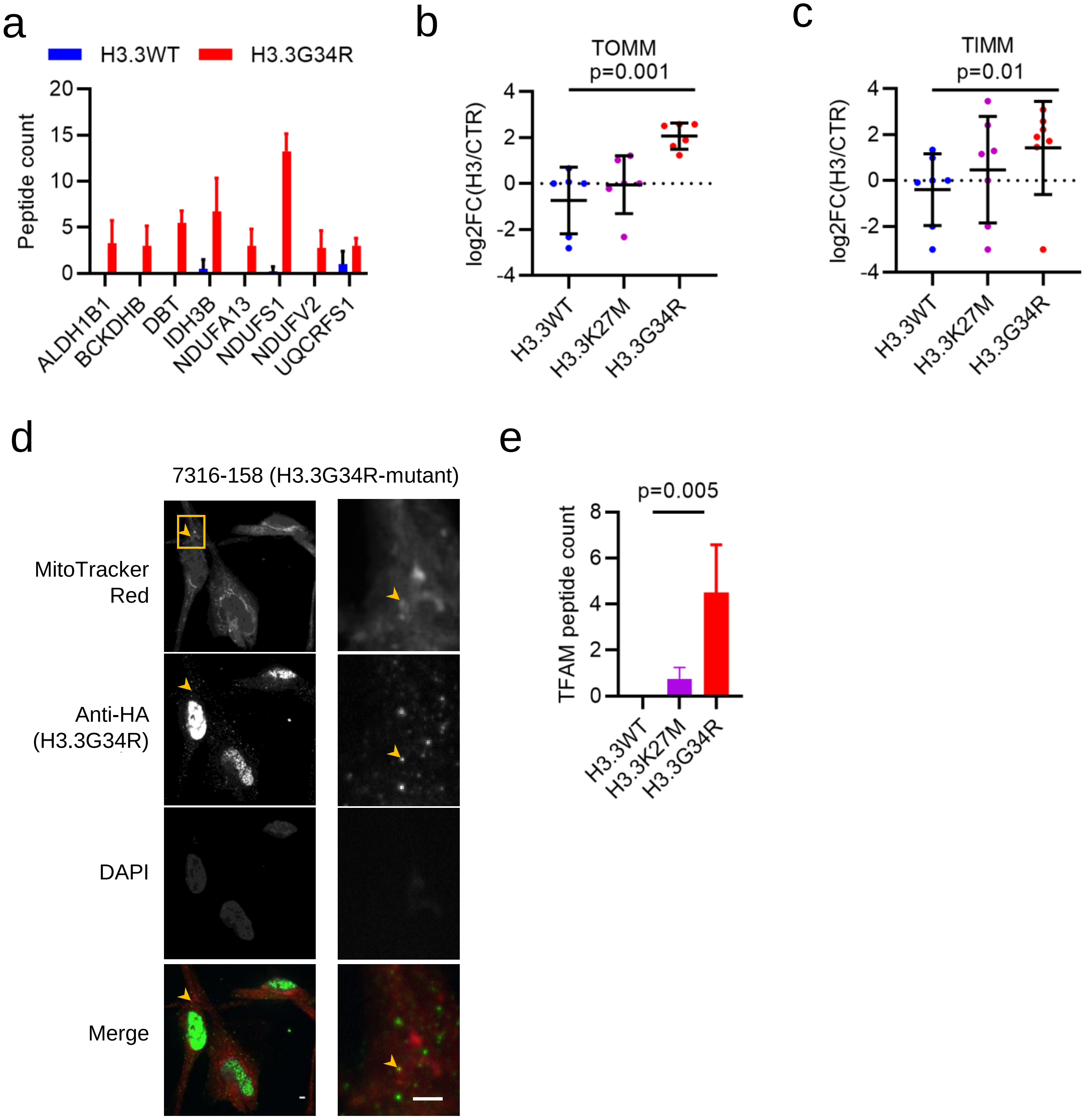

a
H3.3WT
H3.3G34R
Peptide count
ALDH1B1
BCKDHB
DBT
IDH3B
NDUFA13
NDUFS1
NDUFV2
UQCRFS1
b
TOMM
p=0.001
log2FC(H3/CTR)
H3.3WT
H3.3K27M
H3.3G34R
c
TIMM
p=0.01
log2FC(H3/CTR)
H3.3WT
H3.3K27M
H3.3G34R
d
7316-158 (H3.3G34R-mutant)
MitoTracker Red
Anti-HA (H3.3G34R)
DAPI
Merge
e
p=0.005
TFAM peptide count
H3.3WT
H3.3K27M
H3.3G34R


Fig. S3

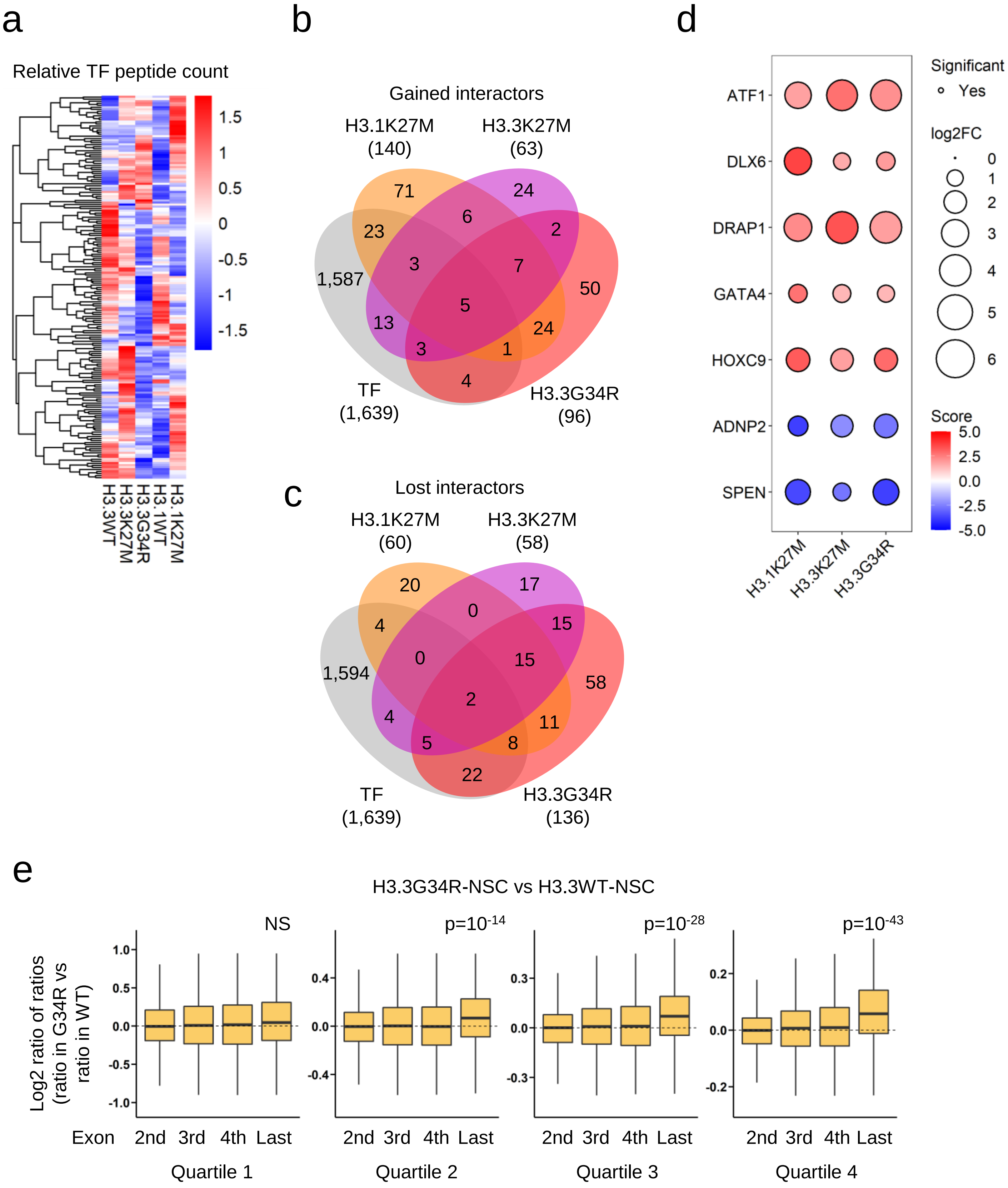
a
Relative TF peptide count
1.5
1
0.5
0
-0.5
-1
-1.5
H3.3WT
H3.3K27M
H3.3G34R
H3.1WT
H3.1K27M
b
Gained interactors
H3.1K27M (140)
H3.3K27M (63)
71
24
6
23
2
3
7
1,587
5
50
13
24
3
1
4
TF (1,639)
H3.3G34R (96)
c
Lost interactors
H3.1K27M (60)
H3.3K27M (58)
20
17
0
4
15
0
15
1,594
58
2
4
11
5
8
22
TF (1,639)
H3.3G34R (136)
d
ATF1
DLX6
DRAP1
GATA4
HOXC9
ADNP2
SPEN
H3.1K27M
H3.3K27M
H3.3G34R
Significant
Yes
log2FC
0
1
2
3
4
5
6
Score
5.0
2.5
0.0
-2.5
-5.0
e
H3.3G34R-NSC vs H3.3WT-NSC
NS
p=10^-14
p=10^-28
p=10^-43
Log2 ratio of ratios (ratio in G34R vs ratio in WT)
Exon
2nd 3rd 4th Last
Quartile 1
Quartile 2
Quartile 3
Quartile 4

Fig. S4

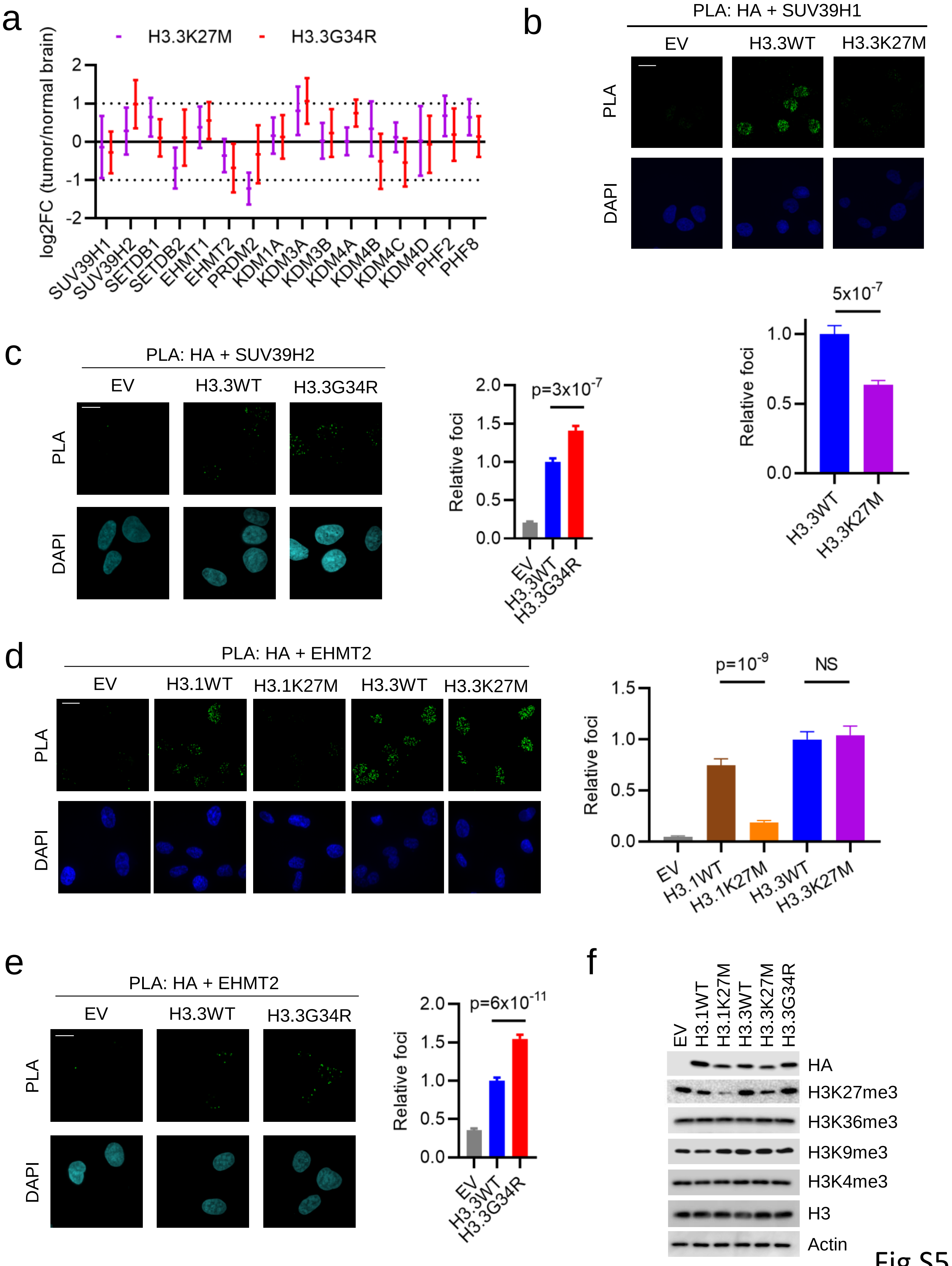

a
H3.3K27M
H3.3G34R
log2FC (tumor/normal brain)
2
1
0
-1
-2
SUV39H1
SUV39H2
SETDB1
SETDB2
EHMT1
EHMT2
PRDM2
KDM1A
KDM3A
KDM3B
KDM4A
KDM4B
KDM4C
KDM4D
PHF2
PHF8
b
PLA: HA + SUV39H1
EV
H3.3WT
H3.3K27M
PLA
DAPI
5x10-7
Relative foci
1.0
0.5
0.0
H3.3WT
H3.3K27M
c
PLA: HA + SUV39H2
EV
H3.3WT
H3.3G34R
PLA
DAPI
p=3x10-7
Relative foci
2.0
1.5
1.0
0.5
0.0
EV
H3.3WT
H3.3G34R
d
PLA: HA + EHMT2
EV
H3.1WT
H3.1K27M
H3.3WT
H3.3K27M
PLA
DAPI
p=10-9
NS
Relative foci
1.5
1.0
0.5
0.0
EV
H3.1WT
H3.1K27M
H3.3WT
H3.3K27M
e
PLA: HA + EHMT2
EV
H3.3WT
H3.3G34R
PLA
DAPI
p=6x10-11
Relative foci
2.0
1.5
1.0
0.5
0.0
EV
H3.3WT
H3.3G34R
f
EV
H3.1WT
H3.1K27M
H3.3WT
H3.3K27M
H3.3G34R
HA
H3K27me3
H3K36me3
H3K9me3
H3K4me3
H3
Actin


Fig S5

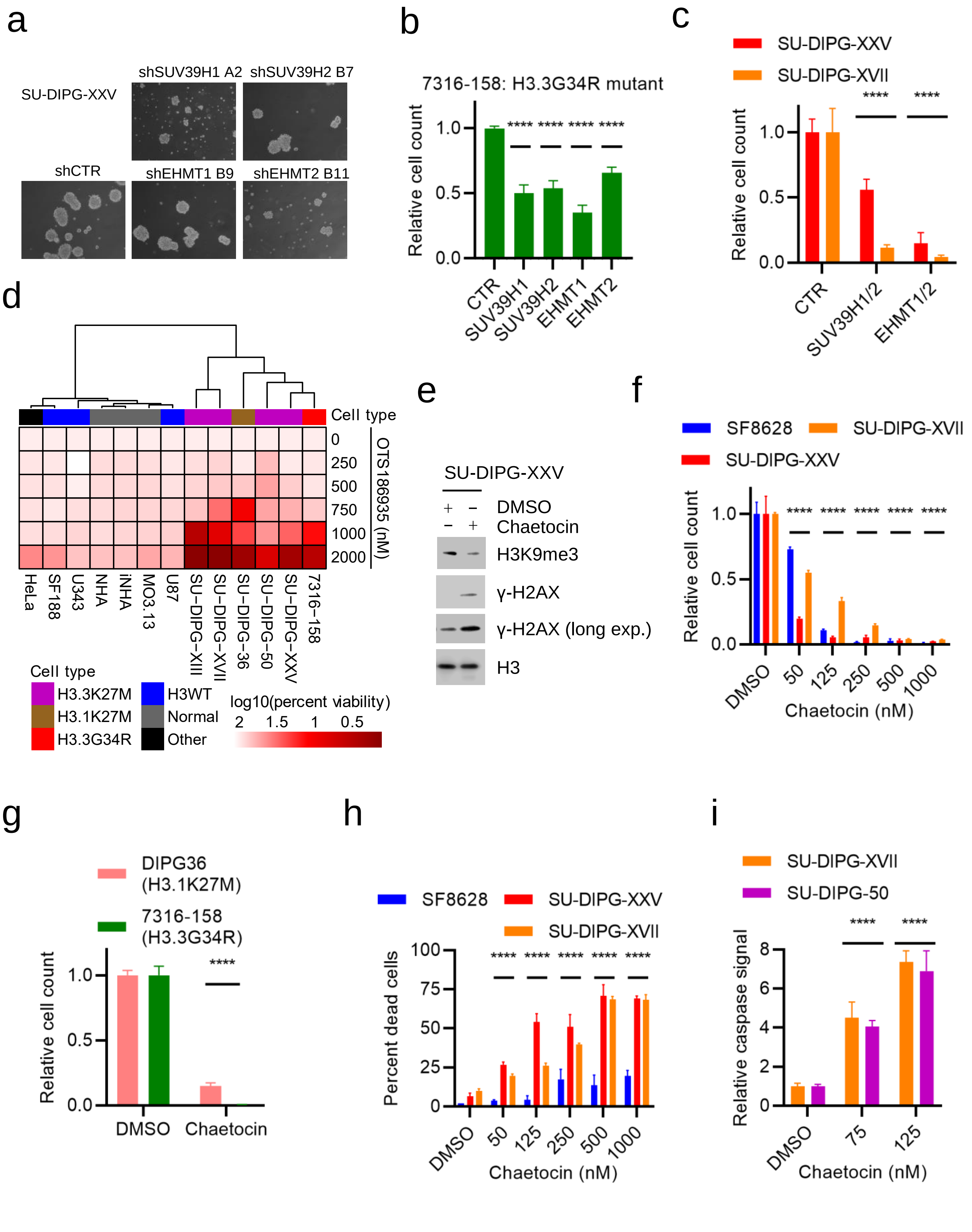

a
SU-DIPG-XXV
shSUV39H1 A2
shSUV39H2 B7
shCTR
shEHMT1 B9
shEHMT2 B11
b
7316-158: H3.3G34R mutant
Relative cell count
CTR
SUV39H1
SUV39H2
EHMT1
EHMT2
c
SU-DIPG-XXV
SU-DIPG-XVII
Relative cell count
CTR
SUV39H1/2
EHMT1/2
d
Cell type
OTS186935 (nM)
HeLa
SF188
U343
NHA
iNHA
MO3.13
U87
SU-DIPG-XIII
SU-DIPG-XVII
SU-DIPG-36
SU-DIPG-50
SU-DIPG-XXV
7316-158
H3.3K27M
H3.1K27M
H3.3G34R
H3WT
Normal
Other
log10(percent viability)
e
SU-DIPG-XXV
DMSO
Chaetocin
H3K9me3
γ-H2AX
γ-H2AX (long exp.)
H3
f
SF8628
SU-DIPG-XVII
SU-DIPG-XXV
Relative cell count
Chaetocin (nM)
g
DIPG36 (H3.1K27M)
7316-158 (H3.3G34R)
Relative cell count
DMSO
Chaetocin
h
SF8628
SU-DIPG-XXV
SU-DIPG-XVII
Percent dead cells
Chaetocin (nM)
i
SU-DIPG-XVII
SU-DIPG-50
Relative caspase signal
Chaetocin (nM)


Fig S6
